# Primordial origin and diversification of plasmids in Lyme disease agent bacteria

**DOI:** 10.1186/s12864-018-4597-x

**Published:** 2018-03-27

**Authors:** Sherwood R. Casjens, Lia Di, Saymon Akther, Emmanuel F. Mongodin, Benjamin J. Luft, Steven E. Schutzer, Claire M. Fraser, Wei-Gang Qiu

**Affiliations:** 1 Division of Microbiology and Immunology, Pathology Department and Biology Department, University of Utah School of Medicine, Salt Lake City, UT USA; 2 Biology Department, University of Utah, Salt Lake City, UT USA; 3 Department of Biology, The Graduate Center, City University of New York, New York, NY USA; 4 Department of Biological Sciences and Center for Translational and Basic Research, Hunter College of the City University of New York, New York, NY USA; 5 Institute for Genome Sciences, University of Maryland School of Medicine, Baltimore, MD USA; 6 Department of Medicine, Health Science Center, Stony Brook University, Stony Brook, NY USA; 7 Department of Medicine, New Jersey Medical School, Rutgers, The State University of New Jersey, Newark, NJ USA; 8 Pathology Department, University of Utah School of Medicine, Room 2200K Emma Eccles Jones Medical Research Building, 15 North Medical Drive East, Salt Lake City, UT 84112 USA; 9 Department of Physiology and Biophysics & Institute for Computational Biomedicine, Weil Cornell Medical College, New York, USA

**Keywords:** *Borreliella*, *Borrelia*, *Burgdorferi*, Lyme disease, Linear plasmid, Circular plasmid, Plasmid evolution

## Abstract

**Background:**

With approximately one-third of their genomes consisting of linear and circular plasmids, the Lyme disease agent cluster of species has the most complex genomes among known bacteria. We report here a comparative analysis of plasmids in eleven *Borreliella* (also known as *Borrelia burgdorferi* sensu lato) species.

**Results:**

We sequenced the complete genomes of two *B. afzelii,* two *B. garinii,* and individual *B. spielmanii*, *B. bissettiae, B. valaisiana* and *B. finlandensis* isolates. These individual isolates carry between seven and sixteen plasmids, and together harbor 99 plasmids. We report here a comparative analysis of these plasmids, along with 70 additional *Borreliella* plasmids available in the public sequence databases. We identify only one new putative plasmid compatibility type (the 30th) among these 169 plasmid sequences, suggesting that all or nearly all such types have now been discovered. We find that the linear plasmids in the non-*B. burgdorferi* species have undergone the same kinds of apparently random, chaotic rearrangements mediated by non-homologous recombination that we previously discovered in *B. burgdorferi*. These rearrangements occurred independently in the different species lineages, and they, along with an expanded chromosomal phylogeny reported here, allow the identification of several whole plasmid transfer events among these species. Phylogenetic analyses of the plasmid partition genes show that a majority of the plasmid compatibility types arose early, most likely before separation of the Lyme agent *Borreliella* and relapsing fever *Borrelia* clades, and this, with occasional cross species plasmid transfers, has resulted in few if any species-specific or geographic region-specific *Borreliella* plasmid types.

**Conclusions:**

The primordial origin and persistent maintenance of the *Borreliella* plasmid types support their functional indispensability as well as evolutionary roles in facilitating genome diversity. The improved resolution of *Borreliella* plasmid phylogeny based on conserved partition-gene clusters will lead to better determination of gene orthology which is essential for prediction of biological function, and it will provide a basis for inferring detailed evolutionary mechanisms of *Borreliella* genomic variability including homologous gene and plasmid exchanges as well as non-homologous rearrangements.

**Electronic supplementary material:**

The online version of this article (10.1186/s12864-018-4597-x) contains supplementary material, which is available to authorized users.

## Background

The growing number of cases of human Lyme and related diseases merits detailed study of the causative pathogens. The bacterial family *Borreliaceae* fam. Nov. contains two clades that are typified by the causative agents human Lyme disease and human relapsing fever (*e. g.*, [1, 2]). Recent taxonomic analyses have argued that the single original *Borrelia* genus that contained these bacteria should be split into two genera, *Borreliella* sp. nov. which contains the Lyme disease agents and close relatives, and *Borrelia* which contains the relapsing fever agents and close relatives [3]. Although the division of the original single *Borrelia* genus into these two genera remains somewhat controversial [4, 5], we choose to use the two-genus nomenclature in this report since it is useful and informative in discussing their evolutionary genomics. Each of these genera contains a number of species, and *Borreliella* currently consists of about twenty species (previously known as “*Borrelia burgdorferi* sensu lato” species, a term we do not use here) that are carried by *Ixodes* ticks in the temperate zones of North America and Eurasia and recently found in South America [6]. Lyme disease is the most common vector-borne human disease in the United States and Europe [7, 8], and it has been associated with seven or eight *Borreliella* species, *B. afzelii, B. bavariensis, B. bissettiae, B. burgdorferi, B. garinii, B. lusitaniae, B. mayonii* and perhaps *B. spielmanii* [9–17] (but also see [18]). These species have highly related chromosomes that are about 900 kbp in length, and they carry numerous plasmids that are more variable in sequence than the chromosome and are variably present in individual isolates [1, 15, 19–21]. These bacteria infect a wide variety of mammals and other vertebrates [22, 23] and exhibit different pathogenicities, tropisms and disease manifestations [24–27]. It is likely that many of these phenotypic differences are the result of differences in plasmid-encoded genes.

Electrophoretic separation of whole *Borreliella* DNAs has invariably shown that these bacteria carry multiple plasmids (*e. g*., [28, 29]). However, multiple linear plasmids in the same cell often have similar size, and circular plasmids are not resolved, so careful (not draft) sequencing is required to delineate all the plasmids in any isolate. Whole genome sequencing of *B. burgdorferi* sensu stricto isolates (hereafter referred to as *B. burgdorferi*) has shown that up to 23 plasmids can be present in a single isolate; the type strain B31’s whole genome sequence contains 21 plasmids [30], and two additional plasmids were shown to have been lost from the sequenced culture [30, 31]. These plasmids carry a large repertoire of genes, many of which encode surface lipoproteins that are important in the detection, prevention and pathogenicity of *Borreliella* infection [32–34]. In addition, a number of plasmid genes are critical for tick transmission and virulence in mice [35–48].

In order to understand the diversity and evolutionary relationships within the *Borreliella* lineage, as well as their human pathogenicity and relationship to the relapsing fever clade, we sequenced the complete genomes, including all the plasmids, of twenty-two isolates from seven *Borreliella* species and a variety of sources [30, 49–53]. These isolates include *B. burgdorferi* and six other species. The plasmids in the fourteen *B. burgdorferi* whole genome sequences include linear replicons that range from 5 to 55 kbp in length and circular replicons between 9 and 61 kbp [20]. We used cosmid library scaffolds to assemble the plasmid sequences, and the plasmids in strain B31 were extensively checked by Southern blot restriction mapping to show that our assembly methods generate accurate plasmid sequences [30, 54]. Multiple stretches of very similar sequences in many of the *Borreliella* plasmids make standard “next generation” short run sequencing methodologies and random library dideoxynucleotide sequencing unable to assemble many of these plasmid sequences. However, recently the plasmids in *B. afzelii* K78 plasmids have been successfully sequenced using highly accurate next generation sequencing and high stringency sequence assembly methods [21, 55], and *B. burgdorferi* strains PAbe and PAli and *B. mayonii* MN14–1420 and MN14–1539 plasmid sequences have been successfully assembled using scaffolds determined by single molecule, real-time (SMRT) long sequence runs (PacBio platform) [15, 21, 55]. The plasmid contents of the above *Borreliella* closed whole genome sequences range from seven to twenty-three plasmids, and in every case these include both linear and circular plasmids. In addition, apparently complete sequences of individual plasmids have been deposited in the public sequence database from *B. garinii* Ip21 [56] and 20047 (BioProject PRJNA350560), *B. bavariensis* PBi [57] and BgVir [58], *B. afzelii* BO23 (BioProject PNJNA359557) and Tom3107 [59], *B. valaisiana* Tom4006 [59], *B. japonica* HO14 (BioProject PRJEB15958) and *B. chilensis* VA1 [60]. We report here a comparative analysis of all available *Borreliella* plasmid sequences.

## Results

### Plasmid types & names

Four hundred and five *Borreliella* plasmid sequences are compared in this study; 236 are from the genomes of fully sequenced *B. burgdorferi* isolates, 141 are from non-*burgdorferi Borreliella* species (hereafter referred to as NBu-*Borreliella* species, Table 1) with fully sequenced genomes, and 28 are anecdotally sequenced plasmids from other NBu-*Borreliella* isolates. (We include the five incompletely assembled strain *B. finlandensis* SV1 lp32–6 and *B. spielmanii* A14S cp32 and cp9 plasmids in this analysis; see below; plasmids from isolates PAbe and PAli were reported [55] after our analysis was completed and are not included.) Two hundred and twenty-two of these plasmids are linear and 183 are circular. Accession numbers for the *Borreliella* plasmids in complete genome sequences can be found in references [15, 21, 30, 49–53, 55], and the remaining plasmid accession numbers are listed in Additional file 1: Table S1. All are available at the Borreliabase world-wide web site http://BorreliaBase.org/ [61]. These plasmids are traditionally named according to the “sequence type” of their paralogous protein family 32 (PFam32) ParA partition proteins; the fact that this very likely defines the plasmid compatibility type has been discussed in detail elsewhere ([19, 30, 62–64] and see below). Analysis of the 169 NBu-*Borreliella* plasmids has not been previously reported, and this study focuses on their relationships.

**Table 1:** Number of plasmids in NBu-*Borreliella* isolates

	Linear plasmids	Circular plasmids
*B. afzelii*		
ACA-1	9	5
BO23	6^a^	2^a^
K78	8	5
PKo	9	8
*B. bissettiae*		
DN127	7	9
*B. finlandensis*		
SV1	5	5
*B. garinii*		
Far04	6	1
PBr	8	3
20047	4^a^	1^a^
*B. japonica*		
HO14	3^a^	1^a^
*B. mayonii*		
MN14–1420	8	7
MN14–1539	7	7
*B. spielmanii*		
A14S	7	6^b^
*B. valaisiana*		
VS116	6	5

^a^Not all plasmids have been identified and sequenced in this isolate

^b^Because the sequences of some circular plasmids in A14S were not closed, it is possible that some cp32s are fused. Thus, the number of different plasmid DNA molecules could be slightly less than this value

**Fig. 1:**
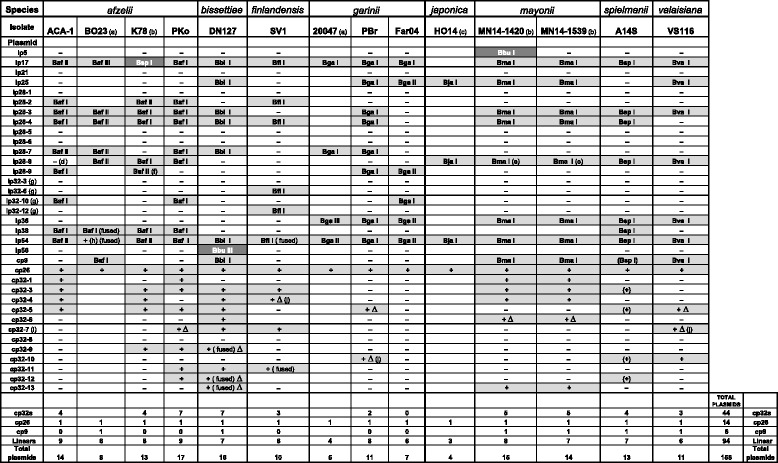
Sequenced plasmids in NBu-*Borreliella* species. The eleven completely sequenced genomes and three partly sequence genomes are shown as columns where shaded cells indicate the plasmid’s presence, “fused” indicates that the indicated plasmids contain apparently intact PFam32 genes of more than one type (see text); and “**∆**”, indicates the presence of a substantial deletion relative to other cp32s. Hyphens (−) denote plasmids that are known not to be present, and blank cells indicate that it is not known if that plasmid type is present. For the linear plasmids and cp9, Roman numerals indicate plasmid organizational subtype (see text and [20] for subtype definitions); here subtypes are named by the first letter of the genus name and first two letters of the species name (e.g., *B. afzelii* subtype I is “Baf I”). Similar subtype numerals in different plasmid types (columns in table) does not imply any relationship, and subtype organizations are always different in the different species except in the three cases that are indicated by dark gray cells (see text). The cp26 plasmids do not exhibit organizational variation and cp32s are too variable, so no subtypes are defined for these plasmids; a “+” indicates that a plasmid of that PFam32 type is present, and parentheses (...) around the subtype name denote the cp9 and four cp32 isolate A14S plasmids whose sequences were not closed. Because the sequences of these plasmids in A14S were not closed, it is possible that some cp32s are fused, so the number of different plasmid DNA molecules is not known precisely in this case. (a) The indicated plasmid sequences from isolates *B. afzelii* BO23 and *B. garinii* 20047 have been deposited in GenBank as closed sequences (Additional file 1: Table S1); it is not known what other plasmids these isolates may carry (S. Bontemps-Gallo, Pers. Com.); (b) these plasmids represent the full complement of plasmids of *B. afzelii* K78 and the two *B. mayonii* isolates; accession numbers are listed in [15, 21], respectively; (c) the plasmid sequences from isolate *B. japonica* HO14 (ATCC51557) are “minimal draft” quality sequences; they are included here because they are the right size to be full plasmid sequences but the details of their sequences should be interpreted with caution. (d) plasmid is very likely present in the original ACA-1 isolate, see text; (e) called lp28–10 in reference [15], see text; (f) called lp28–1 in reference [21], see text; (g) the lp32 plasmids very likely have the same compatibility type as cp32 of the same number, see text; (h) the BO23 lp54 sequence does not include the PFam54 gene array that distinguishes the subtypes of this plasmid; (i) previously named cp32–2 and cp32–7 have the same PFam32 protein type; we use cp32–7 to represent this group; and (j) several kbp of typically linear plasmid sequence replaces at least part of the deleted cp32 DNA

Figure 1 lists the plasmids present in each of the fourteen best understood NBu-*Borreliella* genomes. Nearly all of the sequenced plasmids in these isolates fall into one of the 29 PFam32 types that were previously defined from analysis of the plasmids in fourteen *B. burgdorferi* genomes [20]. These fourteen isolates contain plasmids from 24 of the previously known 29 PFam32 types; they contain no plasmid types lp21, lp28–1 (but see below), lp28–5, lp28–6 or cp32–8. We searched the available NBu-*Borreliella* draft genomes for genes encoding the five “missing” PFam32 protein types and found only one cognate match, a 99% amino acid sequence match to lp28–6 PFam32 protein from Colorado, *B. bissettiae* isolate CO275 (Accession No. OJH14568). It therefore appears that at least lp28–6 is present in some NBu-*Borreliella* isolates. The other four “missing” PFam32 types were not found, but the number of such genome sequencing projects is still rather small so their absence may not be significant.

**Fig. 2:**
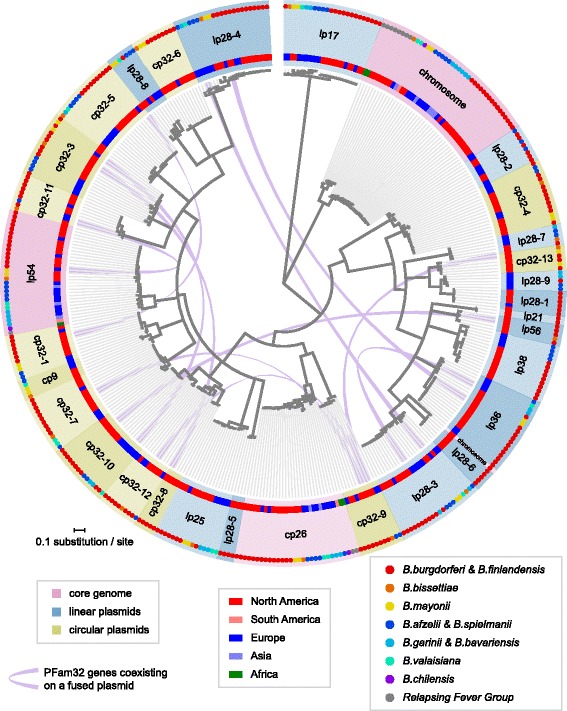
PFam32 gene tree suggests ancestral radiation of plasmid compatibility groups. The midpoint-rooted gene tree (center) is based on a 392-amino acid long alignment of 411 PFam32 homologues from 34 *Borreliella* genomes and selected sequences from 8 relapsing-fever *Borrelia* genomes. The rings, from center outward, indicate the (i) continent of origin of each isolate, (ii) plasmid compatibility type names with different shades of pink, blue, and green for the core genome (chromosome, cp26 and lp54), linear, and circular plasmids, respectively, and (iv) species (colored dots). Lavender central ribbons connect multiple PFam32 alleles found on the same replicon, indicating plasmid fusion events. A vertical PFam32 tree is available as Additional file 1: Figure S1, where two PFam32 homologs (one on lp28–9 from BOL26, another on lp56 from DN127) with positions inconsistent with the genome phylogeny (see Fig. 5 below) – indicative of whole-plasmid transfer – are highlighted. Plasmid types lp5 and some cp9s are not shown in the figure as they do not have a PFam32 partition gene

Only one new type of plasmid-encoded PFam32 protein, carried by some cp9 plasmids, is present in the NBu-*Borreliella* genomes. This is shown in the maximum likelihood PFam32 tree in Fig. 2 and in the similar neighbor-joining tree in Additional file 1: Figure S1. Both trees give identical sets of PFam32 types and show that the cp9 PFam32 proteins form a well-separated branch with strong bootstrap support. This new type is encoded by the circular cp9 (8.5–10.5 kbp) plasmids from *B. afzelii, B. bissettiae, B. mayonii, B. valaisiana* and probably *B. spielmanii* isolates (see cp9 analysis below). We currently lump the cp9 plasmids together even though the *B. burgdorferi* and *B. garinii* versions lack PFam32 genes, because they are otherwise extremely similar. The cp9 PFam32 proteins are 20–25% different in amino acid sequence from their nearest relatives, the cp32–1 PFam32 proteins. The *B. mayonii* isolates both carry a cp32–1 plasmid in addition to such a cp9, so this level of difference is sufficient to allow them to coexist in the same cell. This is consistent with the observation that the previous most closely related pair of PFam32 protein types that we know must have different compatibilities, *B. burgdorferi* JD1 cp32–8 and cp32–12 (for example), are also about 25% different in amino acid sequence [20]. We also note that the lp28–9 plasmid group was previously split from the lp28–1 group (Table S4 in [19, 20]), since their PFam32 genes are 17–18% different, so we include *B. afzelii* K78 “lp28–1” [21] as an lp28–9 in this discussion (Fig. 2 and Additional file 1: Figure S1). In addition, the PFam32 proteins encoded by the two *B. mayonii* plasmids called “lp28–10” [15] are sufficiently closely related to the canonical lp28–8 plasmids that we include them in the latter group in this discussion (the lp28–8 and lp28–10 PFam32 proteins are only 12–15% different; Fig. 2 and Additional file 1: Figure S1). The precise degree of difference among PFam32 proteins that might allow plasmid incompatibility is not known and could well vary in different specific cases, so lp28–1/lp28–9 compatibility and lp28–8/lp28–10 incompatibility remain speculative since no strains are known that carry both members of these pairs. We also note that Fig. 2 and Additional file 1: Figure S1 indicate that especially substantial PFam32 diversity exists *within* several of the plasmid types (e.g., lp28–4 and lp38), and it is possible that these could constitute more than one compatibility type. More experimental work will be required to understand *Borreliella* plasmid compatibility in greater detail. The 169 NBu-*Borreliella* plasmid sequences analyzed here contribute to the current total of 405 plasmids from 28 isolates that bear out the so far inviolate rule that no *Borreliella* cell contains two or more plasmids that encode the same PFam32 type; they thus lend additional credence to the idea that these proteins contribute to plasmid compatibility.

Since only one additional PFam32 type has been found among the 169 NBu-*Borreliella* plasmid sequences from eight species analyzed here, it is likely that additional *Borreliella* plasmid types are rare, geographically restricted, or both. However, we have previously noted that the “orphan” (not in a cluster with the other putative partition genes) PFam32 protein BB_F13 encoded by strain B31 lp28–1 suggests the possible existence of a 31st type (see Additional file 1: Figure S1). We also note that the Fritz-Lipmann Institute *B. afzelii* PKo draft genome sequence (Bioproject PRJNA17057) contains an approximately 13 kbp contig that is not found in our PKo sequence (Bioproject PRJNA68149). It contains a typical cluster of four partition genes that encode PFam49, PFam57, PFam32 and PFam50 proteins (however, the latter is C-terminally truncated), and this PFam32 protein (locus_tag = BAPKO_2556) is about 18% different from its closest relatives, the *B. burgdorferi* lp21 PFam32 proteins. This PKo protein has one close relative in the current database, a 95% identical homologue (locus_tag = SAMN02983004_01117) in a *B. japonica* HO14 draft contig. The latter two proteins form a unique and robust branch in the PFam32 tree (Additional file 1: Figure S1) and thus likely represent a relatively rare 32nd *Borreliella* PFam32 type.

### Plasmid frequencies

Among the eleven NBu-*Borreliella* isolates with completely sequenced genomes, the average number of different plasmids per isolate is 12.8 and the number of different plasmids ranges from seven in *B. garinii* Far04 to seventeen in *B. afzelii* PKo (Fig. 1). These plasmid numbers are minimum values since *Borreliella* plasmids are known to be rather easily lost during laboratory culture [48, 65–68]; indeed, *B. afzelii* strain ACA-1 was previously shown to harbor a plasmid that carries the *vls* cassette region [69] that it is missing from our ACA-1 genome sequence (see below), and our *B. afzelii* PKo genome sequence is missing the novel plasmid mentioned above.

We previously found that among fourteen *B. burgdorferi* isolates the different plasmids are present at different frequencies. For example, plasmids lp17, lp28–4, lp36, lp54 and cp26 were the most common and universally present in all fourteen isolates, and lp28–3, lp38 and cp32–5 were only missing from two, three and three isolates, respectively [20]. Here we find that all eleven of the completely sequenced NBu-*Borreliella* isolates carry lp17, lp54 and cp26. Their next most common plasmids are lp28–3 and lp28–4, each of which is present in nine of the genomes, and lp28–5, lp28–8 and lp36 which are each present in six genomes (Fig. 1). The overall ratios of circular to linear plasmids in the completely sequenced genomes are 0.68 in the NBu-*Borreliella* isolates and 0.92 in the *B. burgdorferi* isolates. This difference is largely due to fewer cp32 plasmids in the former. Cp32s represent 28% of the plasmids in NBu-*Borreliella* and 38% in *B. burgdorferi* isolates, and we note that *B. garinii* Far04 is the only *Borreliella* isolate examined to date that contains no cp32 type plasmid (but it does carry a related plasmid lp32–10 that contains about 28 kbp of cp32-like sequences; see below). Since *B. garinii* PBr has only two cp32s it is possible that the *B. garinii* species in general carry fewer cp32s than the other *Borreliella* species, but this sample is currently too small to draw this as a rigorous conclusion.

### Linear plasmids

**Fig. 3:**
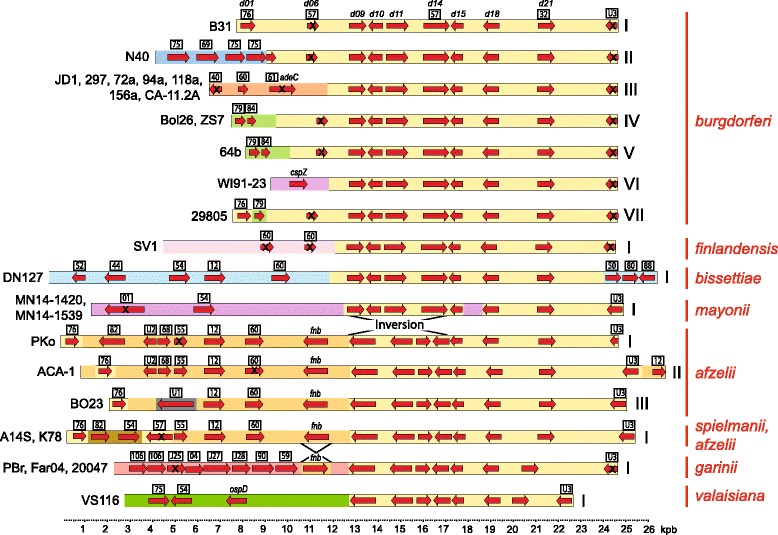
ORF maps of *Borreliella* lp17 plasmids. The horizontal bars represent all the sequenced lp17 plasmids; identical background colors indicate regions of homologous DNA in the different plasmids. The bacterial species and organizational subtypes (see text) are indicated by Roman numerals and on the right (without their species component, see text), and isolates that carry each subtype are indicated on the left. Selected genes are indicated by red arrows, and black “X”s mark pseudogenes. PFam numbers [19, 30] are indicated in the boxes above; “U”s in boxes are proteins for which no intact gene is known in *B. burgdorferi*; “J's” in boxes indicate strain B31 homologous proteins for which there is only one gene in strain B31 (i.e., no PFam number exists); *adeC, cspZ* and *fbn* refer to adenine deaminase, complement regulator-acquiring surface protein Z and fibronectin binding protein encoding genes, respectively; selected strain B31 gene names are indicated above its map. Black lines between plasmids mark the locations of inversions. See Additional file 1: Figure S3B for locations of indels within generally homologous regions (e.g.*,* deletions within B31-like sections of VS116 lp17 and PBr lp17)

**Fig. 4:**
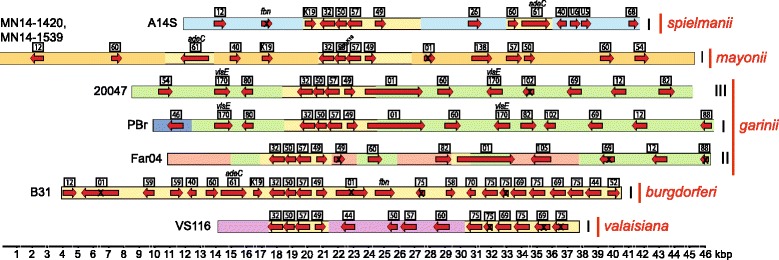
ORF maps of *Borreliella* lp36 plasmids. Plasmids are represented as in Fig. 3, with the same background colors indicating regions of homologous DNA. The organizational subtypes (see text) are indicated by Roman numerals on the right, and isolates that carry each type are indicated at the left

Most linear plasmid PFam32 types in *B. burgdorferi* are present in different isolates as several different “organizational subtypes” [20]. The present analysis shows that this is also true for the NBu-*Borreliella* species. Among the 222 *Borreliella* linear plasmids (99 NBu-*Borreliella* and 123 *B. burgdorferi*, respectively), in only a very small number of cases do cognate plasmids have the same gene organization in more than one species (see below). The vast majority have sequence organizations that are unique to their species. Figures 3 and 4 show, by way of example, comparative open reading frame (ORF) maps of the NBu-*Borreliella* lp17 and lp36 plasmids, respectively. Our previous analysis of *B. burgdorferi* linear plasmids concluded that during their relatively recent evolution they have undergone many apparently random, duplicative and other types of rearrangements mediated by nonhomologous recombination. Genes were often truncated by these rearrangements or after such events redundant genes have undergone random decay that included bp changes and indel formation [19, 20, 30, 64, 70, 71]. The result of this seemingly chaotic evolution is a strikingly low fraction (for a prokaryote) of protein coding DNA in these plasmids, due to the presence of many apparently non-functional pseudogenes and DNA regions that have no long ORFs. NBu-*Borreliella* linear plasmid sequence annotation performed by the JCVI Prokaryotic Annotation Pipeline (www.jcvi.org/cms/research/projects/prokaryotic-annotation-pipeline/overview/) shows that the NBu-*Borreliella* linear plasmids also contain a considerable amount of DNA that appears not to encode proteins. Additional file 1: Figure S2 shows two typical examples, *B. afzelii* PKo lp28–4 and *B. spielmanii* A14S lp36. PKo lp28–4 has 42% protein coding DNA and 44% if unique short genes are included, and A14S lp36 has 31% and 34% protein coding DNA calculated by these two methods, respectively.

Comparative maps of all the NBu-*Borreliella* linear plasmid organizational subtypes are displayed in Additional file 1: Figure S3; the different subtypes within each species are defined and named with Roman numerals as in Casjens et al. [20], and to distinguish the different species here we add the first letter of the genus name and first two letters of the species name (e.g., *B. afzelii* lp17 subtype I is called “lp17 Baf I”. Even though fewer members of each species were analyzed than was done with the fourteen *B. burgdorferi* isolates [20], in the NBu-*Borreliella* species where plasmids of the same type have been completely sequenced from multiple isolates, we find substantial organizational variation in those plasmids (subtypes listed in Fig. 1). In the four *B. afzelii* and *B. garinii* isolates for which the most information is known, ten plasmids, lp17, lp25, lp28–2, lp28–3, lp28–4, lp28–7, lp28–8, lp28–9, lp36 and lp54, display more than one subtype within at least one of these two species (ignoring the fact that BO23 lp38 type Baf I appears to be joined end-to-end to lp54) (Figs. 3, 4 and Additional file 1: Figure S3). The frequencies of plasmid subtypes in the NBu-*Borreliella* genomes are similar to those of *B. burgdorferi*. Interestingly all of the seven *B. mayonii* linear plasmids are the same subtype in both of the sequenced genomes; either this species harbors less plasmid diversity than other *Borreliella* species*,* or the small sample contains two fortuitously closed related *B. mayonii* genomes.

Thus, the nature of the subtype differences among and within the NBu-*Borreliella* species are similar to those described for *B. burgdorferi*, and are characterized by apparently random shuffling of DNA sequence patches among the linear plasmids. Table 2 demonstrates this evolutionary shuffling by listing the varied locations of a number of relatively well-studied genes in the different *Borreliella* genomes. Clearly, these rearrangements did not happen in a common ancestor, but occurred with the same overall chaotic pattern after the separation of the different species. The common forces that drive this peculiar evolution in the different branches of this bacterial lineage remain mysterious.

**Table 2:** Linear plasmid locations of selected NBu-*Borreliella* genes

	Gene^a^	adeC	arp/erpD	bptA	cpsZ	dbpAB^c^	fnb	plzA	ospAB^c^	ospC^c^	ospD	pncA	res-mod1	res-mod2	sagABC DE	vlsE-like^d^	vslE & vls cassettes
	PFam^b^	61	163	99	92	74	none	180	53	none	none	none	1	102–167	none	170	170
*B. burgdorferi*	B31	lp36	lp28–1	lp25	lp28–3	lp54	lp36	–	lp54	cp26	lp38	lp25	lp25, lp28–3	lp56	–	–	lp28–1
*B. bissettiae*	DN127	lp28–7	–	lp25	lp28–3	lp54	lp28–7	–	lp54	cp26	–	lp25	lp25, lp28–3	–	–	–	–
*B. finlandensis*	SV1	–	–	lp28–2	–	lp54^h^	–	lp32–6^f^	lp54^h^	cp26	–	lp28–2	lp32–12^f^	–	–	–	lp32–6
*B. mayonii*	MN14–1420	lp36	–	lp25	–	lp54	–	–	lp54	cp26	lp28–3	lp25	lp25, lp28–4	–	–	–	lp28–8^e^
	MN14–1539	lp36	–	lp25	–	lp54	–	–	lp54	cp26	lp28–3	lp25	lp25, lp28–4	–	–	–	lp28–8^e^
*B. afzelii*	PKo	lp38	–	lp28–2	–	lp54	lp17	–	lp54	cp26	lp32–10 ^f^	lp28–2	lp28–3, lp28–4	lp32–10	lp28–8	–	lp28–8
	K78	lp38	–	lp28–2	–	lp54	lp17	–	lp54	cp26	–	lp28–2	lp28–3, lp28–4	–	lp28–8	–	lp28–8
	ACA-1	lp38	–	lp28–2	–	lp54	lp17	–	lp54	cp26	lp32–10^f^	lp28–2	lp28–3, lp28–4	lp32–10	lp28–8?^j^	–	lp28–8?^j^
	BO23^l^	lp38 ^i^	ND	ND	ND	lp54	lp17	ND	lp54^h^	cp26	ND	ND	lp28–4	ND	lp28–8	ND	lp28–8
*B. garinii*	PBr	lp25	–	lp25	lp28–4	lp54	lp17	lp28–9	lp54	cp26	cp32–10^k^	lp25	lp28–3, lp36	lp28–9 ^f^	–	lp28–4, lp36 ^j^	lp28–3
	Far04	lp25	–	lp25	–	lp54	lp17	–	lp54	cp26	lp32–10	lp25	lp36	lp28–9 ^f^	–	–	lp28–9
	20047^l^	ND	ND	ND	ND	lp54	lp17	ND	lp54	cp26	ND	ND	lp36	ND	ND	lp36 ^j^	ND
*B. spielmanii*	A14S	lp36	–	lp38	lp28–4, lp28–8	lp54	lp17	–	lp54	cp26	–	lp38	lp28–3	–	lp28–8	–	lp28–8
*B. valaisiana*	VS116	right end chrm^f^	–	lp25^j^	lp36 ^f^	lp54	–	–	lp54	cp26	lp17	lp25	lp28–3^f^	cp32–7^k^	lp28–8	lp28–3	lp28–8
*B. japonica*	HO14^l^	ND	ND	lp25	ND	lp54	ND	NC	lp54	cp26	ND	lp25	ND	ND	lp28–8	ND	ND

^a^The linear plasmids that carry selected genes are indicated in the table; only apparently intact genes are indicated; hyphens (−) indicate a plasmid is absent from that completely sequenced genome. Gene names are as follows: *adeC*, adenine deaminase [35]; *arp*, arthritis related protein [134]; *cpsZ*, complement regulatory protein Z [86]; *dbpAB*, decorin binding proteins A and B [135]; *fnb*, fibronectin protein [136–139]; *plzB*, cyclic di-GMP binding protein [140, 141]; *ospAB*, outer surface proteins A and B [142–144]; *ospC*, outer surface protein C [103, 145], *ospD*, outer surface protein D [85, 146]; *pncA*, nicotinamidase [73]; *res-mod1*, restriction-modification protein PFam01 [147, 148]; *res-mod2*, restriction-modification protein PFam102/167 [148]; *sagABCDE*, synthesis of streptolysin S-like toxin [78]; *vlsE-*like, putative lipoproteins that are related VlsE but are encoded at a location not adjacent to *vls* cassette array [19]; *vlsE* and *vls*, variable outer surface protein cassettes and expression locus [149]

^b^*Borreliella* paralogous protein family (see text)

^c^The gene contents of lp54 and cp26 gene content is very constant so only a few examples of their many important genes are shown

^d^*vlsE* homologue not adjacent to *vls* cassette region

^e^Named lp28–10 in the literature but we include them as lp28–8 type plasmids (see text)

^f^A relatively large, apparently truncated gene fragment whose functionality is unknown; there is no intact *adeC* gene in the VS116 genome

^g^Gene is known to have been originally present in this isolate, but its plasmid was lost from the sequenced culture [69]

^h^lp54 is fused to another plasmid (see text); this gene is in the lp54 portion

^i^lp38 is fused to lp54; this gene is in the lp38 portion

^j^Two genes of this sort are present on this plasmid

^k^Unusual circular plasmid location; in B31 lp38-like indel in PBr cp32–10 and B31 lp56-like indel in VS116 cp32–7; see Additional file 1: Figure S8

^l^Plasmid sequences are not complete for this isolate. ND indicates not determined; since all plasmids in this strain have not been sequenced the presence of this gene is not known

#### Lp5, lp21, lp28–1, lp28–5, and lp28–6

No NBu-*Borreliella* was found to contain an lp21, lp28–1, lp28–5 or lp28–6 plasmid, all of which are known in *B. burgdorferi* isolates, and only one other species, *B. mayonii*, has an lp5. Lp5, the smallest of the known *Borreliella* plasmids, is a relatively rare plasmid whose function is unknown and which has only been sequenced from two *B. burgdorferi* isolates and one *B. mayonii* isolate. Additional file 1: Figure S3A shows aligned reading frame maps of these three plasmids. The relationships among these three very similar plasmids is discussed below in terms of possible horizontal plasmid transfer; however, we note here that all three are extremely similar and the only putative plasmid replication/partition gene on the *B. mayonii* lp5, which encodes a Pfam57 protein, has a frameshift in codon 116 that likely inactivates its function; perhaps its role can be substituted by a paralogue encoded by another plasmid [63].

#### Lp17

**Fig. 5:**
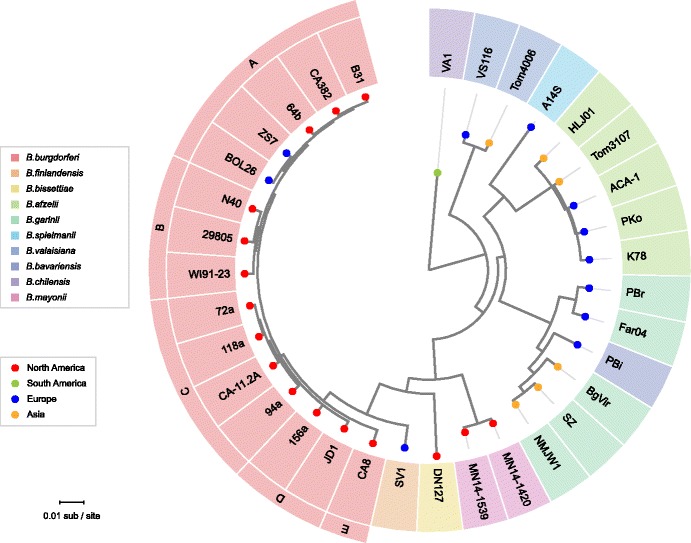
*Borreliella* chromosome phylogeny. It is based on a concatenated 144,891-long protein sequence alignment of 450 single-copy orthologues universally present in all *Borreliella* genomes (see Methods) and is rooted with orthologues from relapsing-fever *Borrelia* genomes (not shown). The four previously designated *B. burgdorferi* chromosomal “SNP types” A, B, C and D [1] and a new type E are indicated outside the circle in the same color box as the cognate strain names. Isolate names are shown within the outer ring, and species designations of the genomes are indicated by color in the outer ring. Continental origins of the isolates are indicated by colored dots at the tips

On the other hand, all the NBu-*Borreliella* complete genome sequences contain an lp17 plasmid. The fourteen *B. burgdorferi* lp17s are present as seven different organizational subtypes, and curiously the major differences among these types are all different left end sequences [20]. Figure 3 and Additional file 1: Figure S3B show that the NBu-*Borreliella* lp17s also have a variety of different sequences at their left ends, and several also have shorter substitutions at their right ends. Within species, the two *B. mayonii* lp17s are nearly identical, as are the three *B. garinii* plasmids. The four *B. afzelii* lp17s, on the other hand are present as four different subtypes (the *B. afzelii* K78 lp17 is discussed in more detail below). The *B. afzelii, B. garinii, B. spielmanii* and *B. valaisiana* lp17s all have different organizations, but they all contain the same inversion of a central gene cluster (homologues of *bb_d09* through *bb_d14*) relative to other lp17s (Fig. 3), supporting the idea derived from chromosome phylogeny ([1] and see Fig. 5 below) that these species have a common ancestor after their separation from the branch that contains *B. burgdorferi, B. bissettiae, B. finlandensis* and *B. mayonii* [1, 15, 49, 72].

#### Lp25

Several of the NBu-*Borreliella* species carry lp25 plasmids, but the three *B. afzelii* and single *B. finlandensis* SV1 and *B. spielmanii* A14S complete genomes, do not. The two *B. mayonii* lp25 plasmids are nearly identical, but the two *B. garinii* lp25s have substantial differences and represent two different subtypes (Additional file 1: Figure S3C). Interestingly, in spite of major organizational differences, all these lp25s carry a *pncA* gene ([73, 74] and Additional file 1: Figure S3C), a *bptA* gene [75] and a *bb_e17* homologue. The lack of lp25-borne *pncA, bptA* and *bb_e17* genes in the *B. afzelii, B. finlandensis* and *B. spielmanii* genomes is compensated for their presence on lp28–2 in *B. afzelii* and *B. finlandensis* and lp38 in *B. spielmanii* (Table 2). We also note that the *B. valaisiana* lp25 carries a 10.5 kbp imperfect direct repeat (the two arms of the repeat are about 98% identical) that includes these three genes as well as the partition gene cluster; but one of the *pncA* reading frames is not intact.

#### Lp28–2

Only four NBu-*Borreliella* genomes, three *B. afzelii* and *B. finlandensis* SV1 genomes, harbor an lp28–2, all of which have very substantial organizational differences from *B. burgdorferi* lp28–2s (Additional file 1: Figure S3D). The three *B. afzelii* plasmids have two very different left and right ends and clearly represent two subtypes. Curiously, the SV1 lp28–2 has a left end sequence that is organizationally the same as the *B. afzelii* ACA-1 lp28–2 suggesting a possible horizontal exchange of that part of one of these two plasmids. As mentioned above, these *B. afzelii* and *B. finlandensis* lp28–2s each carry a *pncA, bptA* and *bb_e17* gene.

#### Lp28–3

Among the NBu-*Borreliella* genomes only *B. finlandensis* lacks an lp28–3 plasmid, and again these are present as a number of different organizational subtypes that have substantial inter-species differences (Additional file 1: Figure S3E). The two *B. mayonii* lp28–3s are very closely related, and the four *B. afzelii* plasmids comprise two subtypes. The *B. garinii* PBr lp28–3 is unique among the NBu-*Borreliella* genomes in that it carries a set of *vls* cassettes; the *B. burgdorferi* Bol26 lp28–3 also carries *vls* cassettes but, other than similar PFam32 genes it has little else in common with the PBr lp28–3. *B. valaisiana* lp28–3s large size is unusual at over 82 kbp; this is in part due to 21 kbp of rather highly degraded cp32-like DNA (Additional file 1: Figure S3F).

#### Lp28–4

Only *B. valaisiana* VS116 and *B. garinii* Far04 do not contain an lp28–4. Again, each species contains unique subtypes (Additional file 1: Figure S3G). The two *B. mayonii* plasmids are very closely related, and the four *B. afzelii* plasmids comprise two subtypes (curiously the *B. afzelii* BO23 subtype II is similar to the other cognate *B. afzelii* plasmids, but appears at the current stage of sequence assembly to be circularly permuted relative to the others; not shown).

#### Lp28–7

Among the NBu-*Borreliella* genomes, lp28–7 plasmids are only present in the *B. afzelii, B. bissettiae* and *B. garinii* genomes. Again, in every case the different species contain unique subtypes (Additional file 1: Figure S3D). The three *B. afzelii* plasmids form three subtypes, and the two *B. garinii* PBr and 20047 lp28–7s form another subtype. The two *B. garinii* lp28–7s and the *B. afzelii* ACA-1 and BO23 lp28–7s all contain the 21 kbp cluster of tightly packed genes that are homologous to the section of *B. burgdorferi* B31 lp28–2 that has features of bacteriophage virion assembly operons [76, 77], but nearly half of the parallel *B. afzelii* PKo lp28–7 cluster is deleted and partly replaced by other sequences.

#### Lp28–8

Linear plasmid lp28–8 is not found in the *B. bissettiae, B. finlandensis* or *B. garinii* genomes, but each of the other NBu-*Borreliella* genomes contains a unique lp28–8 subtype (Additional file 1: Figure S3H). Again, the two *B. mayonii* plasmids are very closely related to one another (we include the *B. mayonii* “lp28–10s” in the lp28–8 type, see above), and the *B. afzelii* PKo and K78 lp28–8 s have the same subtype. All nine known lp28–8 s carry a set of *vls* cassettes, and *B. afzelii* strain ACA-1 was previously shown to harbor a plasmid that carries the *vls* cassette region [69], but this plasmid was not present in our ACA-1 sequence. The partial ACA-1 sequence reported by Wang et al. [69] contains 775 bp adjacent to the *vls* cassette region whose sequence is essentially identical to parallel sequences in the *B. afzelii* PKo and K78 lp28–8 plasmids, suggesting that the missing ACA-1 plasmid may well be an lp28–8 even though its PFam32 gene was not sequenced. In addition, all the NBu-*Borreliella* lp28–8 s except those in *B. mayonii* carry the *sagA/B/C/D/E/F* gene cluster that is predicted to encode a streptolysin S-like precursor peptide and the enzymes that convert it into a toxin [78]. No other plasmid types have been found to carry these genes. Our previous analysis showed that the *sag* genes are absent from all of the *B. burgdorferi* and *B. bavariensis* isolates tested and are rare if present at all in *B. garinii*, but they are common in *B. afzelii, B. spielmanii* and *B. valaisiana* isolates and may also be present in *B. lusitaniae* isolates [78].

#### Lp28–9

Two *B. afzelii* and two *B. garinii* genomes contain lp28–9 plasmids, and they constitute four organizational subtypes (Additional file 1: Figure S3D). The *B. garinii* Far04 lp28–9 carries a set of *vls* cassettes, and the *B. garinii* PBr and *B. afzelii* ACA-1 and K78 plasmids contain the 21 kbp cluster of *B. burgdorferi* B31 lp28–2 bacteriophage-like genes (above) [76, 77].

#### Linear cp32-type plasmids

Among the fourteen *B. burgdorferi* isolates with sequenced genomes, two, 72a and 118a, carry a *linear* plasmid with a cp32–3 type PFam32 gene. These plasmids were named lp32–3, and they only carry a small number of genes that appear to be derived from cp32 plasmids in addition to the cp32–3-like four-gene partition cluster (Additional file 1: Figure S3I). Five *linear* plasmids are present in the NBu-*Borreliella* genomes that have PFam32 genes of the cp32–6, − 10 and − 12 types (Fig. 1). *B. finlandensis* SV1 carries lp32–6 and lp32–12 plasmids whose PFam32 genes are closely related to those of cp32–6 and − 12, respectively, and one *B. garinii* isolate (Far04) and two *B. afzelii* isolates (PKo and ACA-1) carry lp32–10s that have a cp32–10-like PFam32 gene (Fig. 2 and Additional file 1: Figure S1). The *B. burgdorferi* lp32–3s and SV1 lp32–6 carry a *vls* cassette region, but have little else in common. The *B. afzelii* lp32–10s, like the lp32–3s, have little cp32-like DNA beyond their partition gene clusters, but the SV1 lp32–6 and − 12 plasmids and Far04 lp32–10 have much more cp32-like DNA, about 27, 32 and 28 kbp, respectively. The relatively long approximately 49 kbp SV1 lp32–6 plasmid includes a nearly complete cp32 that appears to have integrated by a recombination event in its homologue of the B31 cp32–1 *bb_p04* gene. Genes *bb_p05-p09* (about 3 kbp) of the expected cp32-like sequence are missing, but some of this sequence could be at the left plasmid terminus that might not have been reached by the sequencing project. This plasmid’s sequence was not closed, so it is not certain that it is linear, but the presence of a *vls* cassette region at the right end of the contig is strongly suggestive that it is linear because this cassette array only occurs at near ends of linear plasmids in all cases where its location is known. In the 52.5 kbp long SV1 lp32–12, the recombination occurred in the homologue of the B31 cp32–1 *bb_P22* gene and sequences from there to *bb_P17* are not present. Its *BSV1_x50* protein is 73% identical to strain *B. burgdorferi* CA12 OspE [79, 80] and 69% identical to B31 ErpP [81], and *BSV1_x51* protein is 53% identical to N40 Erp27 [82]. The Far04 lp32–10 has apparently incorporated cp32 DNA sequences twice. It contains one approximately 23 kbp section that is homologous to B31 cp32–1 genes *bb_p11* through *bb_p41* that contains the cp32–10 PFam32 gene and second approximately 6.5 kbp section that is homologous to the B31 cp32–1 region from about *bb_p23* through *bb_p32*. The Far04 lp32–10 contains no intact *erp-*like gene but does carry homologues of *ospD* and *cspZ,* genes that are present on other linear plasmids in other strains [83–86] (Table 2).

#### Lp36

Lp36 plasmids are present in *B. garinii, B. mayonii, B. spielmanii* and *B. valaisiana*, where again each subtype is limited to a single species (Fig. 4 and Additional file 1: Figure S3J). The two *B. mayonii* lp36s are very closely related, while the three *B. garinii* plasmids represent three subtypes. The *B. garinii* 20047 and PBr lp36s are very similar except for a 2.5 kbp replacement at the left end and the different positions of a transposase (PFam82) gene. The *adeC* (adenine deaminase), *bb_k32* (fibronectin binding protein) and *bb_k46* genes of *B. burgdorferi* B31 lp36 are known to be important for *B. burgdorferi* virulence [35, 45], and in the NBu-*Borreliella* species *adeC* is present on lp36 in the *B. mayonii* and *B. spielmanii* genomes, but is found on lp25, lp28–7 or lp38 plasmids in other species; only a relatively large *adeC* fragment is found at the right end of the chromosome in *B. valaisiana* VS116 (Table 2). Fibronectin binding protein genes are absent from the *B. finlandensis* and *B. mayonii* genomes and only a fragment is present on *B. valaisiana* VS116 lp28–3, but they are present on lp28–7 in *B. bissettiae* DN127 and lp17 in the *B. afzelii, B. garinii* and *B. spielmanii* genomes (Table 2). Curiously, among the NBu-*Borreliella* genomes, a gene closely related to *bb_k46* is only found in *B. valaisiana* VS116, and it contains a reading frame disruption; more distantly related members of this PFam75 family are found on plasmids in the *B. afzelii, B. garinii* and *B. spielmanii* genomes.

#### Lp38

Among the NBu-*Borreliella* genomes, lp38 plasmids are only found in the *B. afzelii* and *B. spielmanii* genome sequences, and their PFam32 proteins form a separate branch that is 10–12% different from the *B. burgdorferi* lp38 PFam32s (Fig. 2 and Additional file 1: Figure S1). All four of the *B. afzelii* plasmids are closely related and belong to the same subtype (Additional file 1: Figure S3K); however, the BO23 lp38 is annotated to be fused end-to-end to lp54 and as such constitutes a second subtype. The *B. afzelii* and *B. spielmanii* lp38 plasmids are very different from each other except for the partition gene cluster, and, uniquely, lp38 carries the only *pncA, bb_e17* and *bptA* genes in *B. spielmanii* A14S and the *adeC* gene in *B. afzelii* (above).

#### Lp54

The *Borreliella* lp54 plasmid sequences are quite uniform in their gene content and organization with a few differences at their left ends, in the *ospA-ospB* gene cluster, in the gene *bb_a53*-*bb_a54* region and in the tandem cluster of PFam54 protein encoding genes near the right end. In addition, we find two major rearrangements involving lp54s; the *B. finlandensis* SV1 lp54 contains an integrated cp32–11 (see below) and the BO23 lp54 is reported to be fused end-to-end with an lp38 (above). Samuels et al. [87] showed previously by Southern analyses that in the strains they analyzed *B. afzelii* and *B. garinii* lp54s are several kbp longer than those of *B. burgdorferi*, and the genome sequences show that *B. afzelii, B. garinii* and *B. spielmanii* lp54s have approximately 3 kbp left end extensions relative to *B. burgdorferi* lp54s (not shown). These extensions are similar in these three species and carry two genes that encode a PFam52 putative lipoprotein (the terminal gene) and a PFam60 putative lipoprotein (penultimate gene). The reported *B. japonica* HO14 lp54 has a different 2 kbp left end extension that encodes a PFam12 putative lipoprotein and a PFam82 (transposase) protein, and *B. chilensis* lp54 has a third type (also about 2 kbp) of left end extension that encodes two unique proteins of unknown function. In all three left-end extension types the first gene internal to the extension is a homologue of *B. burgdorferi* B31 terminal gene *bb_a01*, a homologue of the PFam48 outer membrane channel forming protein P13 [88, 89]. The leftmost gene in other NBu-*Borreliella* lp54s is in all cases a *B. burgdorferi* B31 *bb_a01* homologue, but between the telomere and this gene *B. bissettiae* DN127, *B. finlandensis* SV1 and the two *B. mayonii* strain lp54s all have multiple short insertions relative to the *B. burgdorferi* plasmids that add up to about 100, 200 and 500 bp of “extra” apparently non-protein coding DNA, respectively, compared to B31 lp54 which has 653 bp between the telomere tip and *bb_a01* [51, 90]. The *B. valaisiana* lp54 left end has (different) indels in this region, but the distance from its projected telomere location and the first gene (also a *bb_a01* homologue) is about the same as B31 lp54. The *ospA-ospB* gene region contains one or two genes in different *Borreliella* isolates. All the completely sequenced NBu-*Borreliella* lp54s examined except those of *B. garinii* lp54s contain two genes in this cluster. *B. garinii* isolates PBr, Far04 and 20047 have only one gene. We also note that close relatives of *B. garinii, B. bavariensis* isolates PBi and BgVir have two genes here [57], and Yabuki et al. [91] found that a large fraction of Japanese *B. garinii* isolates have two genes in this cluster. In addition, *B. bissettiae* DN127 lp54 is missing ~ 760 bp relative to *B. burgdorferi* lp54s so that it does not have homologues to *B. burgdorferi* genes *bb_a53* and *bb_a54*; interestingly, *B. valaisiana* isolates VS116 and Tom4006 are identical to one another in this region and have a slightly different deletion relative to *B. burgdorferi* lp54 that removes the same two genes. Finally, the *B. chilensis* lp54 has several organizational (indel) differences from *B. burgdorferi* lp54 in the 15–17.5 kbp region and an approximately 650 bp insertion at about 21.6 kbp (B31 lp54 coordinates); the latter is predicted to encode a unique 124 amino acid protein (locus_tag OY14_04520). Thus, these lp54 organizational differences correlate with individual species or particular groups of species.

We have previously shown that the PFam54 gene cluster, which encodes proteins that affect host complement function and/or bind host plasminogen [92–95], is the most variable region of the lp54 plasmids, and we have described these variations in in terms of gene losses and gains [96, 97]. These clusters are quite variable in gene content and number both within and between species, and since our previous analysis nine more NBu-*Borreliella* lp54 sequences have been reported, making any organizational difference correlations (or lack thereof) in these species much more significant. Additional file 1: Figure S4 shows that there are four conserved PFam54 genes make up the constant outside portions of the cluster and that there are 28 different gene types in the variable region (ignoring small, apparently pseudogene fragments that are present in some cases). The number of genes in the variable region ranges from two in *B. chilensis* to six in some *B. afzelii, B. bissettiae, B. garinii* and *B. valaisiana* isolates. We note that the *B. afzelii* PKo lp54 has been sequenced twice and one version (accession No. CP000396) has two additional genes in the cluster that are identical to *B. bavariensis* PBi lp54 genes BGA71 and BGA72. If this sequence is accurate, it indicates that these genes have likely been lost from the other sequenced PKo culture and the maximum number of variable genes in the cluster would be eight. The PFam54 cluster differences create two lp54 subtypes in *B. afzelii, B. bavariensi*s and *B. garinii* and three in *B. burgdorferi*. In no case is the whole array of PFam54 genes the same in isolates from different species, but the *B. afzelii* ACA-1 and *B. spielmanii* A14S arrays are very similar except for a 37% difference in encoded protein sequences between A14S *BpsA14S_a0067* and its *B. afzelii* orthologues (Additional file 1: Figure S4). Essentially identical arrays are often present in different members of the same species. However, similar individual variable region genes occur in different species in several cases. For example, members of this gene type exemplified by *B. afzelii* PKo gene *BafPKo_a0062* also occur in *B. spielmanii, B. valaisiana, B. japonica* and *B. chilensis* PFam54 clusters, genes similar to *B. afzelii BafPKo_a0066* are present in *B. garinii, B. spielmanii* and *B. bavariensis*, and genes of the same type as *B. burgdorferi* B31 *bb_a68* (*cspA*) are present in *B. bissettiae, B. finlandensis* and *B. mayonii* lp54s. The top four species in Additional file 1: Figure S4, *B. burgdorferi, B. bissettiae, B. finlandensis* and *B. mayonii*, have no variable gene types in common with the remainder of the *Borreliella* species in the figure, which agrees with other analyses that suggest that these groups form two separate lineages ([1] and see Fig. 5 below). Single nucleotide polymorphism (SNP) tree analysis showed that the known whole lp54 plasmids have not exchanged between species [1].

#### Lp56

Lp56 is a relatively uncommon plasmid. Four of the fourteen *B. burgdorferi* genomes harbor this plasmid type (one of which, the strain B31 lp56 contains an integrated cp32), but *B. bissettiae* DN127 is the only NBu-*Borreliella* genome that harbors an lp56, and its lp56 is very similar to the lp56 present in *B. burgdorferi* CA.11_2A (Additional file 1: Figure S3L). This is discussed in more detail below as a possible recent whole plasmid horizontal transfer.

#### Linear plasmid-like sequences at chromosome ends

*Borreliella* chromosomes are very constant except for some differences which have been described previously in the ribosomal RNA gene region [1, 21, 98], in the gene *bb_0524* region [1], and at the right end of *B. burgdorferi* chromosomes [19, 20, 29, 99, 100]. Our previous work showed that most NBu-*Borreliella* chromosomes terminate at about the same location [29]. Only *B. japonica* IKA2 left end appeared to be extended with 15–20 kbp of extra plasmid-related DNA, but its sequence is not known. Additional file 1: Figure S displays ORF maps of the sequences at the ends of all the reported NBu-*Borreliella* chromosomes. Except for the *B. mayonii* and *B. valaisiana* VS116 chromosome, the left end chromosome sequences all terminate at about the same place. The *mayonii* chromosomes have a left end extension about 1100 bp long that carries a gene that encodes a PFam12 protein whose closest relatives are on *B. burgdorferi* plasmids. The *B. valaisiana* left end extension contains only small ORFs that include closely related homologues of fragments of VS116 *BvaVS116_h0023* and *_e0057* proteins (encoded by VS116 plasmids lp28–3 and lp25, respectively). Uniquely, both the known *B. valaisiana* left ends lack a homologue of the *B. burgdorferi* B31 left terminal chromosomal gene *bb_001*, but this gene is present as the terminal gene at these chromosomes’ right ends (Additional file 1: Figure S5B), suggesting a past termini switching event.

Some *B. burgdorferi* right chromosomal ends have variable lengths (up to about 20 kbp) of “extra” plasmid-like DNA compared to the shortest chromosomes, and restriction mapping suggested that several other *Borreliella* species chromosomes terminate at about the same location as the minimal *B. burgdorferi* chromosome (e.g., strain N40) a few hundred bp to the right of the last common chromosomal gene *bb_843* ([20] and references therein). Genome sequences show that all the NBu-*Borreliella* chromosomal right ends do indeed terminate just beyond the right end of their *bb_843* gene homologues (Additional file 1: Figure S5B), with the exception of *B. valaisiana*, which has right end extensions of about 8600 and 8900 bp in the two strains. These extensions are very similar except for a few small indels and an approximately 550 bp inversion. The internal regions these extensions have about 4800 bp that is about 90% identical to *B. afzelii* plasmid lp38. These are a new type of linear plasmid-like right end chromosome extension that contains PFam26, PFam40 and PFam47 genes as well as the *bb_001* homologue and several pseudogenes. Once again, no left or right end extensions are the same in members of different species, so all these rearrangements appear to have occurred after evolutionary separation of these species.

### Circular plasmids

#### Cp9

The NBu-*Borreliella* species also carry circular plasmids that are similar to *B. burgdorferi* cp9, cp26 and cp32 plasmids. We noted above that, unlike the *B. burgdorferi* cp9s, some of the NBu-*Borreliella* cp9 plasmids encode a unique type PFam32 protein. The cp9s in different species are all of different organizational subtypes (Additional file 1: Figure S6), but all have substantial regions of homology (the functions of the encoded proteins are largely not known). Interestingly the *B. burgdorferi* cp9s all carry the PFam95 *eppA* gene [101], but among the NBu-*Borreliella* complete genomes, only *B. bissettiae* DN127 cp9 carries a close relative of this gene (more distantly related “BapA” PFam95 proteins are encoded by cp32 plasmids in several of these genomes).

#### Cp26

The NBu-*Borreliella* genomes all include a cp26 plasmid, which is the only plasmid that is essential for growth in culture in *B. burgdorferi* [102]. As has been previously observed, all NBu-*Borreliella* cp26s gene contents that the same as those of *B. burgdorferi* [1, 20, 103]. This plasmid encodes the important surface protein OspC, and although the rest of the cp26 sequences have diverged only a few percent, *ospC* is probably the most variable single-copy gene in the *Borreliella* genome [1], and it has undergone a substantial number of horizontal exchange events [104–109]. Single nucleotide polymorphism (SNP) tree analysis showed that the known whole cp26 plasmids have not exchanged between species [1].

#### Cp32s

The *Borreliella* cp32 plasmids are prophages, and some phage been shown to produce phage virions that can in turn deliver these plasmids to other cells [76, 77, 110, 111]. All *B. burgdorferi* isolates carry at least five of these homologous and syntenic, but divergent plasmids, and NBu-*Borreliella* isolates also usually carry multiple cp32 plasmid types (Fig. 1). As mentioned above, the two complete *B. garinii* genomes carry fewer cp32s than the other *Borreliella* species that have been examined. *B. garinii* Far04 has no cp32 plasmid, and PBr has two, both of which have apparent defects. PBr cp32–10 has a 3.8 kbp deletion in the putative virion assembly gene cluster that is replaced by about 10.5 kbp of DNA that is quite similar to sequences found on *B. burgdorferi* strain B31 lp38, VS116 lp17 and MN14–1420 lp28–4. PBr cp32–5 has two smaller rearrangements, a deletion that truncates *BgaPbr_a0033*, the PFam49 gene in the partition gene cluster, and an approximately 1.4 kbp deletion that removes two of the three tandem paralogous genes that are homologues of the B31 cp32–1 PFam148 genes *bb_p03*, *_p04* and *_p05.* MN14–1420 cp32–13, VS116 cp32–7 and SV1 cp32–11 also have apparent reductions of this ancient gene triplication to one or two genes, but such a deletion is not present in any of the *B. burgdorferi* cp32s. Since the function of these genes is unknown (they are postulated to lie in the phage virion assembly gene operon [76, 77, 111]), it is not known whether such deletions might impair any phage function.

The other NBu-*Borreliella* genomes have larger cp32 complements than *B. garinii*. *B. burgdorferi* isolates average 6.6 cp32s, and the three *B. afzeli*i genomes average five cp32s, *B. bissettiae* DN127 has seven (that have nine PFam32 genes, see below), *B. finlandensis* SV1 has three (with four PFam32 genes due to a cp32 fusion event), *B. valaisiana* VS116 has three, and the two *B. mayonii* isolates each have five (Fig. 1). *B. spielmanii* A14S apparently has four cp32s but they have not been completely assembled and remain in draft status (see Additional file 1: Figure S7 for analysis of the incompletely assembled A14S cp32 contigs). Most of the NBu-*Borreliella* cp32s appear to be intact but 12 (~ 13%) of their 90 completely assembled cp32s have undergone some type of rearrangement (18% of the 120 completely assembled *B. burgdorferi* cp32s have rearrangements). Additional file 1: Figures S8 and S9 show the locations the various NBu-*Borreliella* cp32 rearrangements; none of them except the PBr cp32–5 PFam49 gene truncation (above) affect the partition gene cluster, which is expected to be required for long term plasmid maintenance and/or stability (reveiwed by [62, 63]) and none affect the *erp/elp/ospEF/Bap* gene containing variable regions 3 and 4 (the latter defined in [19]). VS116 cp32–7 and SV1 cp32–4 have substantial insertions of linear plasmid-like DNA that are similar to parts of *B. burgdorferi* 94a lp56 and *B. finlandensis* SV1 lp28–4, respectively; the former also has a large approximately 8 kbp inverted duplication that includes the partition genes. DN127 contains a complex 66 kbp cp32-related circular plasmid named “cp32-quad” that contains four different partial cp32 sequences that include three different apparently intact PFam32 genes that correspond to cp32–9, − 12 and − 13 types (see Additional file 1: Figure S9). Like the *B. burgdorferi* cp32s, NBu-*Borreliella* cp32s show diversity within species; however, the two *B. mayonii* genomes each contain six cp32s and each pair of cognate cp32s is virtually identical, once more emphasizing that these isolates are extremely similar. On the other hand, five different cp32s are present in more than one of the three *B. afzelii* isolates ACA-1, PKo and K78, and in only one case, ACA-1 and K78 cp32–3, do the cognate plasmids from different isolates have similar *mlp* and/or *erp* gene regions (see Additional file 1: Figure S8), but even in that case they have an approximately one kbp region of substantial difference that encodes the proteins encoded by genes *BafACA1_s16* and *BafK78_s015*.

Interestingly, in *B. finlandensis* SV1 cp32–11 is integrated into lp54 to form an 83,377 bp hybrid linear plasmid that is diagrammed in Additional file 1: Figure S10A. The integrated cp32–11 is nearly full-length but contains a deletion in the *B. burgdorferi* B31 *bb_p03*, *_p04* and *_p05* paralogue cluster (above) that fuses the N-terminal end of the B31 cp32–1 *bb_p04* homologue to the C-terminal portion of the *bb_P05* homologue (annotated as *bsv1_a103*). Genes were disrupted on both plasmids by the integration event, which occurred within the B31 lp54 gene *bb_a65* homologue and the B31 cp32–1 gene *bb_p06* homologue with no apparent deletion or insertion of extraneous nucleotides in either plasmid at the site of integration. The deduced fusion sites have no apparent sequence similarity, so integration was achieved by nonhomologous recombination (see Additional file 1: Figure S10B). This cp32 integration location is different from the location at which cp32–10 integrated into lp56 in strain B31 [30] or the cp32 integrations that formed the lp32s above. The facts that the integration sites on these cp32s are all different and that different genes were disrupted in the target plasmids in all cases suggests that these cp32 integration events occurred at random locations by non-homologous recombination.

## Discussion

### Inter-species whole plasmid mobility

#### Chromosome tree correlates with species

Horizontal transfer of small regions of DNA has been identified as being fairly common in the *Borreliella* species [104–109]. However, as described above, linear plasmid organizational types are typically limited to individual species, indicating that whole plasmid exchange between these species is not common. In order to better understand the evolutionary relationships between the chromosome and plasmids, as well as to search for possible whole plasmid exchanges between species, we constructed a maximum likelihood phylogenetic chromosome tree based on 450 proteins that are universally present as single copies in *Borreliella* chromosomes (Fig. 5). This expanded tree’s major branches correlate with the different *Borreliella* species, and the tree has the same branching order for the different species as the previously published chromosomal SNP tree for a subset of these species, and it supports the idea that *B. finlandensis* and *B. mayoni*i are more closely related to *B. burgdorferi* than to the other *Borreliella* species. The chromosomal tree also indicates that the newly sequenced isolates BgVir, SZ and NMJW1 are in fact *B. bavariensis* as has been previously noted by Margos et al. [112] and Gatzmann et al. [113]. This tree agrees perfectly with the *B. burgdorferi* chromosomal SNP types A, B, C and D that we defined previously [1], and shows that the chromosome of newly sequenced *B. burgdorferi* isolate CA382 is type A and that of CA8 forms a new type, which we designate type E. We also note that the *B. Chilensis* chromosome lies on a deep branch that separates it from all the other *Borreliella* species. Comparison of plasmid sequence relationships with the chromosome tree allowed the discovery of four examples of possible of whole linear plasmid horizontal transfer events between species lineages.

#### Lp5 exchange

*B. mayonii* MN14–1420 is the only NBu-*Borreliella* isolate known to carry the uncommon lp5 plasmid, and only *B. burgdorferi* isolates B31 and WI91–23 were previously known to carry it (Additional file 1: Figure S3A). In the 5225 bp that have been sequenced in all three lp5s, MN14–1420 lp5 has 16 single bp differences from B31 lp5 but only one bp difference from WI91–23 lp5 (the WI91–23 lp5 sequence is missing 24 bp at the left end and has extra 2 bp at the right end relative to B31 and MN14–1420 lp5s). The remainder of the *B. mayonii* and *B. burgdorferi* genomes are substantially more different than this. For example, the *B. burgdorfer*i B31 and WI91–23 chromosomes (99.5% identical to one another) are both 94.8% identical to the *B. mayonii* chromosomes (which are themselves > 99.99% identical). Thus, this appears to be an example of quite recent cross-species transfer of the whole lp5 plasmid between these two strains from mid-west US. The direction of this putative transfer between the WI91–23 *B. burgdorferi* chromosomal type B lineage and *B. mayonii,* cannot be deduced unambiguously, but the alternate hypothesis, lp5 was present in a common ancestor, would require very different mutation rates in the two *B. burgdorferi* lineages A and B and the less parsimonious losses from the lineages of the twelve other *B. burgdorferi* isolates, the one *B. finlandensis* isolate, the one *B. bissettiae* isolate and one of the *B. mayonii* isolates*.*

#### Lp56 exchange

The ~ 29.5 kbp lp56 plasmids that are present in *B. bissettiae* DN127 and *B. burgdorferi* CA.11_2A are 99.97% identical with only eight single bp differences and no indels between the 29,418 bps that have been sequenced in both plasmids (the left and right ends of CA.11_2A sequence are missing ten and twenty terminal bps, respectively, relative to the DN127 sequence). *B. burgdorferi* WI91–23 lp56 is the only other sequenced plasmid that has a long region of synteny with the CA.11_2A and DN127 lp56 plasmids, and it has a several kbp replacement at its left end relative to them (see Figure S20 in [20]). The homologous DNA between 11 and 25 kbp of DN127 lp56 is only 93.8% identical to the parallel WI91–23 region (with no indels larger than 15 bp) (Additional file 1: Figure S3L). In addition, the CA.11_2A and WI91–23 chromosomes are only 0.48% different while they are 1.73% and 1.74% different from DN127, respectively [1]. This unusually close lp56 similarity very strongly suggest its recent transfer between *B. bissettiae* and the *B. burgdorferi* CA.11_2A chromosomal type C lineage. We also note that these lp56s all carry a long region of synteny with *B. burgdorferi* linear plasmid lp28–2 that includes its genes *bb_g10* through *bb_g28* which have been proposed by Eggers et al. [110] to constitute a phage virion assembly gene operon, so it is plausible that these lp56 plasmids are prophages and that their horizontal transfer could be mediated by phage virions.

#### Lp17 exchange

*B. spielmanii* A14S and *B. afzelii* K78 lp17s are members of the same unique subtype which we call lp17 Bsp I (Additional file 1: Figure S3B), but their sequences are not as close as the lp5 or lp56 cases discussed above. A14S lp17 has two short indels relative to K78 lp17, both of which are changes in the number of repeats in a short tandem sequence repeat array; one is the deletion in A14S of four of the six and half 21 bp tandem imperfect repeats (centered at about bp 100 in A14S) that are present in K78 lp17, and the other is 11 more copies in A14S than in K78 of the 21 bp repeat in the different imperfect repeat array that begins at bp 20,884 in 14S. If these repeat differences are removed, the entire A14S and K78 lp17 sequences are 99.2% identical (ignoring K78 lp17’s 29 extra bp at its right end and 23 extra bp at its left end due to failure of the A14S sequence to reach the telomeres; we also note that it has not been shown that these differences are not sequence assembly errors). The lp17 subtype Bsp I plasmids are organizationally different from the PKo, ACA-1 and BO23 lp17 subtypes Baf I, II and III, respectively (Fig. 3 and Additional file 1: Figure S3B). The region between 6500 and 19,000 bp of A14S lp17 avoids the above repeat regions and terminal organizational differences and provides 12,500 bp of syntenic DNA for all of these four subtypes of lp17. In this region A14S and K78 lp17s are 0.6% different from one another, but range from 1.2 to 1.8% different from the other three *B. afzelii* lp17s (lp17 subtypes Baf I, II and III range from 0.4% to 1.8% different from one another). Thus, in addition to the major terminal organizational differences, the large central syntenic regions in the A14S and K78 lp17s are 2- to 3-fold less divergent from one another than they are from the other *B. afzelii* lp17s. On the other hand, the chromosomes of K78, PKo and ACA-1 are all 0.220–0.254% different from one another and 0.534–0.535% different from the chromosome of A14S. Thus, the K78 chromosome fits perfectly in *B. afzelii* and not *B. spielmanii*. These observations indicate that a *B. afzelii* K78-like predecessor donated its lp17 to the *B. spielmanii* A14S lineage or vice versa. Light will be shed on this directionality by additional *B. spielmanii* lp17 sequences - will they be unique or K78-like? The latter would be parsimonious with the *B. spielmanii* to *B. afzelii* direction.

#### Lp28–9 exchange

The European *B. burgdorferi* isolate Bol26 is the only one of the sixteen sequenced genomes of this species (including recently reported strains PAbe and PAli [55]) that carries an lp28–9, while two of three European *B. afzelii* genomes and both European *B. garinii* genomes carry an lp28–9. Its absence from the fifteen other *B. burgdorferi* genomes suggests that Bol26 might have obtained lp28–9 from one of these European species. The Bol26 lp28–9 organization is most similar to its counterparts in *B. afzelii* strains ACA-1 and K78 (Additional file 1: Figure S3D), where it is 87% and 84% identical, respectively, to these plasmids over their common 18,000 bp central regions. The ACA-1 and K78 plasmids are 95% identical over this region. There are also organizational differences among the Bbu I, Baf I and Baf II lp28–9 subtypes. Thus, while it is parsimonious to conclude that Bol29 obtained its lp28–9 from a European *B. afzelii*, if this is true it must have occurred long enough ago to have allowed the observed sequence divergence and terminal organizational changes occur.

### Primordial origin of *Borreliella* plasmids

#### PFam32 protein phylogeny

To further investigate the origin and evolution of *Borreliella* plasmids, we first analyzed the phylogenic tree of their PFam32 proteins shown in Fig. 2. These PFam32 proteins are both orthologous and paralogous; the orthologues are similar in amino acid sequence across species due to direct, non-duplicative descent, and the paralogues are divergent within the same genome due to ancestral duplication events. The 30 types of PFam32 orthologues found on characterized *Borreliella* plasmids (depicted with different shading on the outer ring in Fig. 2) are delineated based on long tree branches as well on the criterion that no two orthologues should coexist within the same genome [19, 20, 64]. Three conclusions can be drawn from this analysis of the PFam32 protein phylogeny. First, the PFam32 tree supports ancestral, primordial radiation of many of the plasmid types. This conclusion is a more parsimonious explanation of the observation that the large majority of plasmid types exist in multiple *Borreliella* species across the species and continents, than the alternative hypothesis in which the different plasmid types have invaded multiple *Borreliella* species independently after they arose in one of the species (especially with the observation above that exchange of whole plasmids between species is rare). Most of the plasmid types appear to be present around the world. Only the lp28–9 PFam32 type is unique to Eurasian *Borreliella* genomes, while PFam32 plasmid types lp21, lp28–1, lp28–5, lp28–6, and cp32–8 (and lp5 which has no PFam32 gene) have to date been found only in North American isolates. However, these findings, especially the Eurasian deficiencies, could be due to the smaller sample sizes of the Eurasian isolates. We also note that although the lp28–1 type is currently unique to North America and the lp28–9 type is unique to Eurasia, the available data do not rule out the possibility that these two groups represent a single compatibility group (above). The ancestral existence of numerous major plasmid groups in the most recent common ancestor of all *Borreliella* species suggests essential roles of these plasmids in *Borreliella* as a biological species complex.

The second conclusion drawn from the PFam32 tree in Fig. 2 is that plasmid fusion, apparently mediated by homologous and/or non-homologous recombination, occurs occasionally in *Borreliella* genomes. The coexistence of two PFam32 paralogues of different type on a single plasmid is indicative of recent plasmid fusion event, since such fusions are not shared by closely related strains. The following plasmids with fusions of this type are present in four Eurasian isolates: lp36/lp28–3 fusion in *B. garinii* Far04 that has an “orphan” lp28–3 PFam32 gene (not in a partition cluster), lp28–3/lp28–4/lp38 fusion in *B. valaisiana* VS116 (at this point only the lp28–3 PFam32 gene is intact), cp32–3/cp32–10 fusion in *B. burgdorferi* ZS7 and *B. finlandensis* lp54/cp32–11 in SV1. We also note that an lp38/lp54 fusion has been reported for European *B. afzelii* BO32, but it is not included in this analysis since its genome sequence appears is still in draft status. Six such fusion plasmids are present in North American strains as follows: cp32–9/cp32–12/cp32–13 in the cp32-quad plasmid of *B. bissettiae* DN127, and the following fusions in *B. burgdorferi*: lp56/cp32–10 in B31, lp36/lp28–4 in CA11-2A, cp32–7/cp32–9 in 118a, cp32–3/cp32–8 in 64b, cp32–1/cp32–5 in JD1. These plasmid fusions that carry more than one PFam32 gene are indicated by lavender ribbons in Fig. 2. In addition, the lp32–3, − 6, − 10 and − 12 plasmids in which linear plasmids appear to have fused with cp32s and subsequently lost their linear plasmid PFam32 genes are limited to *B. burgdorferi* chromosomal lineage C, the relatively closely related species *B. afzelii* and *B. garinii*, and *B. finlandensis*, respectively. These findings suggest that these fusion plasmids arose relatively recently in specific lineages.

On the other hand, these fused plasmids are not always shared by closely related isolates suggesting that the fusion events likely occurred after strain divergence. For example, (i) among the *B. burgdorferi* type A chromosome isolates the lp56/cp32–10 fusion is found in B31, PAli and PAbe, but not in 64b, ZS7 or Bol26, while different cp32 fusions are present in 64b and ZS7 that are not in the other type A isolates, and (ii) the lp36/lp28–4 fusion in CA11-2A and cp32–7/cp32–9 fusion in 118a are not present in other *B. burgdorferi* type 3 chromosome isolates. Past fusions may or may not resolve rapidly. We postulate that homologous recombination-mediated fusions form frequently and can resolve rapidly. Indeed, recently Margos et al. [55] reported that their culture of *B. burgdorferi* B31 (B31 NRZ) carries a cp32–1/cp32–5 fusion, while the cp32–5 portion was not present in the MedImmune culture of B31 that was originally sequenced [51, 70]. This suggests that either a resolution of this fused plasmid occurred and the cp32–5 portion was lost in the latter culture, or a fusion of the two plasmids occurred in the former culture. This apparently rapid fusion and resolution of cp32 plasmids likely reflects the fact that homologous recombination can mediate both in the substantial regions of similar sequence that cp32s contain.

Plasmids similar to the fused lp56 present in the New York isolate B31 were identified by Palmer et al. [114] in two (Connecticut) isolates out of eleven New England *B. burgdorferi* isolates tested. In addition, lp56 plasmids that are essentially identical to B31 lp56 are present in the sequenced genomes of two *B. burgdorferi* type A isolates PAbe and PAli isolated from two German patients that likely were infected in North America [55, 115]. The fact that these three plasmids are almost certainly all the result of a single past fusion event [20, 30], suggests that reversal of this fusion is not rapid. In the B31 lp56/cp32–10 and SV1 lp54/cp32–11 cases where the parental sequences can be deduced at the fusion site, the fusions are clearly mediated by non-homologous recombination (Additional file 1: Figure S10B and [30, 70]). Such fusions cannot be easily reversed by homologous recombination and so are not expected to resolve rapidly or precisely.

Third, the PFam32 tree supports of the putative horizontal transfer (by lysogeny or transduction) of whole-plasmids discussed above. The donor and recipient genomes of these events can be suggested by comparing the PFam32 tree (Fig. 2 and Additional file 1: Figure S1) and the chromosome tree (Fig. 5). For example, the latter tree indicates that *B. spielmanii* is an outgroup of the *B. afzelii* clade; however, the PFam32 orthologue on *B. spielmanii* A14S lp17 groups with its *B. afzelii* lp17 orthologues, supporting the displacement (since all current isolates have an lp17) of an lp17 plasmid between an *B. spielmanii* and *B. afzelii* (above). Similarly, inconsistent PFam32 groupings indicate an uptake of lp28–9 by *B. burgdorferi* Bol26 from a *B. afzelii* strain (above), and an uptake of an lp56 by *B. bissettiae* DN127 from a *B. burgdorferi* strain (above).

#### Early PFam32 divergence before Borreliella-Borrelia divergence

We further examined these ideas by incorporating relapsing fever (RF) PFam32 proteins into our analysis. Less information has been reported about RF plasmids than about *Borreliella* plasmids, but both branches carry numerous plasmids, and at least some cognate plasmids have mosaic relationships within the RF lineage, similar to those of the *Borreliella* plasmids ([116, 117] and our unpublished observations). The RF plasmids that have been analyzed have surprisingly little gene content in common with *Borreliella* plasmids other than partition genes and a small number of other scattered homologous genes [116, 117]. The exception to this that we are aware of is the presence of cp32-like plasmids in RF isolates, some of which are as follows: *B. hermsii* HS1 plasmid cp28 [118, 119], *B. duttonii* Ly plasmids lp26, lp27 and lp28 [117], *B. miyamotoi* CT13–2396 plasmids cp1, cp2, cp3, cp4 and cp5 [120] and *B. coriaceae* Co53 unnamed plasmids with accession numbers CP005764 and CP005752. Like the *Borreliella* cp32s they have sizes that are largely in the 25–30 kbp range with the exception of several in the 13–19 kbp range (truncated versions of cp32s are also present in *Borreliella* isolates ([20] and references therein)). These RF plasmids have overall synteny with the *Borreliella* cp32s, but their nucleotide and encoded protein sequences are rather distantly related to their *Borreliella* homologues. In addition, database searches with one of the important phage virion structural genes carried by the cp32 prophage plasmids, the putative head portal proteins of PFam146, identified syntenic but smaller draft sequence contigs in *B. hispanica, B. crocidurae, B. persica,* and *B. parkeri* genomes. It thus appears that, as in the *Borreliella* isolates, multiple cp32-like plasmids can inhabit individual bacteria, and they are quite widely distributed across the RF lineage. When we included 45 plasmid-encoded PFam32 proteins from RF isolates *B. hermsii* HS1 [119], *B. miyamotoi* CT13–2396 [120], *B. crocidur*ae Achem (Bioproject PRJNA85135), *B. turicatae* BET5EL [121], *B. duttonii* Ly and *B. recurrentis* A1 [117] in the neighbor-joining PFam32 tree, they represent at least 24 different sequence types, none of which coincide with any of the *Borreliella* PFam32 types discussed above, yet nearly all of them group robustly *inside* the *Borreliella* PFam32 tree (Additional file 1: Figure S11). And as expected none of these RF strains carry more than one plasmid with any given PFam32 type. We conclude that the origin of *Borreliella* plasmids must predate the divergence of the *Borreliella* and RF *Borrelia* clades. The alternate explanations, massive horizontal PFam32 gene transfer or loss, are much less parsimonious.

#### Partition gene cluster phylogeny

**Fig. 6:**
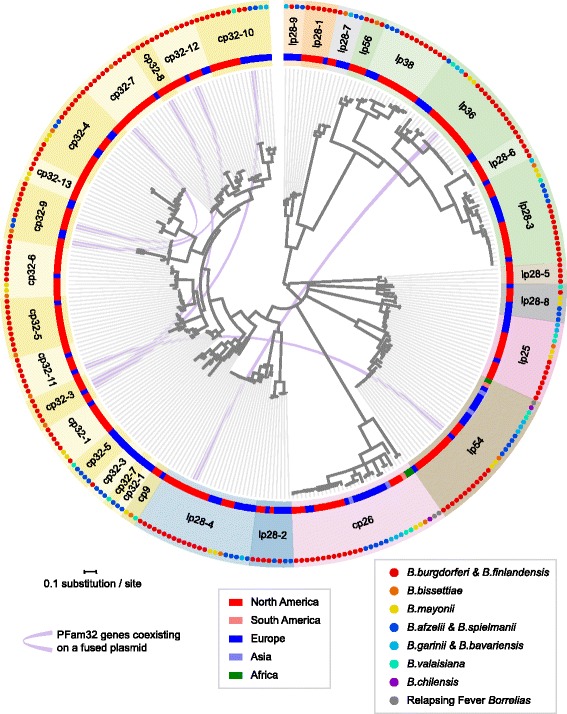
Plasmid super-groups revealed by plasmid partition gene clusters. The midpoint-rooted gene tree (center) is based on a 1327 amino acid long concatenated alignment of sequences of four plasmid partition proteins (including PFam57/62, Pfam50, PFam32, and Pfam49). This tree shows a monophyly of all cp32 + cp9 plasmids (left-side, shades of yellow in outer ring). Other monophyletic plasmid super-groups separated by similarly deep branches include (counter-clockwise from bottom) lp28–4/lp28–2, cp26, lp54, lp25, lp28–8, lp28–5, lp28–3/lp28–6/lp36/lp38/lp56, lp28–7, and lp28–1/lp28–9. Plasmids not shown in the figure (lp5, lp17 and lp21) do not have a full set of all four partition genes. A web-interactive version of the tree is available at BorreliaBase website (under the “Replicons” tab)

Finally, to further resolve the history of plasmid evolution in *Borreliella*, we built a phylogeny of the *Borreliella* plasmids that harbor a complete set of plasmid partition genes [20, 30, 64]. These partition gene clusters consist of four, usually tandemly arranged genes that encode protein members of the PFam57/62, PFam32, PFam50, and PFam49 families. The total length of this seven concatenated alignment is 1327 amino acids (including gaps), more than four times as long as the 392 amino acid alignment of PFam32 sequences alone, so it provides more information for phylogenetic reconstruction. The tree derived from these concatenated sequences is shown in Fig. 6. Trees based on a single locus can be misleading if a locus has undergone rare recombination events, and phylogenetic trees based on sequences of other individual cp32 proteins, including Pfam80, PFam165, PFam144, and PFam96, show large inconsistencies (trees not shown; see also references [19, 84]). Including more partition genes minimizes the effect of such misleading information. Unlike the PFam32 tree (Fig. 2), the partition gene cluster protein tree (Fig. 6) shows monophyly of all cp32 + cp9 plasmids, suggesting a single common origin for them, with the cp32s separated from the cp9s by a relatively shallow branch. Other major monophyletic plasmid groups separated by deep branches from the others include cp26, lp25, lp28–1/lp28–9, lp28–2/lp28–4, lp28–3/lp28–6/lp36/lp38/lp56, lp28–7, lp28–5, lp28–8 and lp54. The unlisted plasmids (lp5, lp17, and lp21), which are the smallest linear plasmids, do not have a full set of all four partition genes and so were not included in this analysis. These plasmid relationships will provide a phylogenetic framework for understanding the history of plasmid rearrangements, between-genome plasmid and gene exchanges, as well as gene gains and losses.

## Conclusions

There are currently 405 *Borreliella* complete plasmid sequences in the nucleotide sequence database. We previously analyzed and compared the 236 *B. burgdorferi* plasmids [19, 20, 30], and here we analyzed the remaining 169 plasmid sequences from eight other *Borreliella* species, *B. afzelii, B. bavariensis, B. bissettiae, B. garinii, B. finlandensis, B. spielmanii*, *B. valaisiana* and *B. chilensis.* This study of complete genomes from seven new species discovered only one new type of PFam32 putative plasmid compatibility type in addition to the 29 types that were previously known, suggesting that all or nearly all of the types have now been identified. However, since there are currently fourteen additional named or proposed *Borreliella* species whose plasmid sequences have not yet been examined, it remains quite possible that they might harbor new plasmid types. Only a few of the plasmid PFam32 types are *apparently* species-specific at our current knowledge state; lp21, lp28–5, lp28–6 and cp32–8 are found only in *B. burgdorferi* strains, and no types are restricted to other *Borreliella* species. In addition, only a few PFam32 types are currently restricted to the Eastern or Western Hemispheres; the lp28–9 type is unique to Eurasian *Borreliella* genomes, while plasmid types lp28–1, lp28–5, lp28–6 and cp32–8 have been found only in North American isolates, but these apparent restrictions may be due to the fact numerous *Borreliella* species from both hemispheres have not yet been analyzed. During their evolution, the linear plasmids in NBu-*Borreliella* species have undergone the same kinds of chaotic rearrangements mediated by non-homologous recombination that we have previously described in the *B. burgdorferi* lineage. These rearrangements have occurred independently in each of the different species lineages, and they allowed the identification several rare whole plasmid transfer events between species.

Phylogenetic analyses of the plasmid partition genes showed that a majority of the different plasmid PFam32 types arose early, most likely before separation of the current *Borreliella* and *Borrelia* species, and this, perhaps with occasional cross lineage plasmid transfers, has resulted in few if any species-specific PFam32 plasmid types. Indeed, it is possible that the invention, invasion and/or domestication (from prophages in some cases) of these plasmids occurred in evolutionarily rapid succession and is the key evolutionary event that both defined the species and made the *Borreliacae* successful global parasites. Phylogenetic analysis of the four-gene plasmid partition clusters showed that the cp9 and cp32 plasmid PFam32 genes share a single common origin that is distinct from the other, mostly linear *Borreliella* plasmids, and it allowed the deduction of an evolutionary hierarchy of related groups among the linear plasmids. The improved resolution of *Borreliella* chromosome and plasmid phylogeny will lead to better determination of gene orthology, which is essential for prediction of biological function, and it will provide a basis to infer detailed mechanisms of *Borreliella* genomic variability including homologous gene and plasmid exchanges as well as non-homologous rearrangements.

The persistent maintenance of all major plasmid groups in *Borreliella* among its global lineages investigated here suggests not only their functional indispensability but also their evolutionary role in enhancing genome diversity and species evolvability. Indeed, being a closed genome with few gains and losses of gene families since their divergence [1], much of the genome variability and dynamism in *Borreliella* are mediated by plasmids, including whole-plasmid exchanges and structural rearrangements described here and earlier [20], higher sequence diversity as a results of increased homologous recombination and diversifying selection at surface-antigen loci on plasmids [104, 122] and in situ recombination at *vls* cassettes locus [123]. Disrupting plasmid partition and maintenance may therefore be an effective means to halt the local transmission cycles and geographic expansion of the *Borreliella* endemics worldwide. Similarly, knowledge of the variability of the *Borreliella* genome and its many plasmids will be informative in understanding host-range infectivity factors and varying host clinical responses.

## Methods

### Plasmid types and subtypes

We have previously described the sources of the isolates whose genomes have been sequenced and the methods used for sequence determination [1, 20]. Plasmid types and subtypes were determined and analyzed as described previously [20]. After determination of the encoded PFam32 protein type using ClustalX neighbor-joining tree analysis (Additional file 1: Figure S1), BLASTn [124], as well as DNA Strider [125] (usually initially at a window stringency setting of 17 out of 23 bp identities) and Gephard ([126] dot plot plasmid comparisons were used to identify groups of very similar plasmids (subtypes) that do not have large indels relative to one another. Nucleotide and protein sequence alignments were performed by BLASTn and BLASTp [124], ClustalX [127] and DNA Strider [125]. Neighbor-joining trees were created by ClustalX [127], and the resulting trees were drawn by NJPlot (http://pbil.univ-lyon1.fr/software/njplot.html). Maximum likelihood trees were inferred with RAxML [128]. Determination of percent identity between chromosomes was determined through reference-independent whole-chromosome alignments using MUGSY [129] as described in reference [1].

### Chromosome tree

We identified 450 single-copy orthologues on the main chromosome (list available from author WGQ) using homolog identification by pairwise BLASTp and homolog clustering by CD-HIT [130]. Orthologous protein sequences were aligned using MUSCLE [131, 132]. Using the BIOALN utility of the BbWrapper toolkit (https://metacpan.org/release/ROCKY/Bio-BPWrapper-1.02), the protein alignments were concatenated and an approximate maximum likelihood tree was inferred using FastTree [133]. The resulting tree was re-rooted using orthologues from relapsing-fever *Borrelia* genomes and branches with low bootstrap support (< 0.8, if any) were removed using the BIOTREE utility of the BbWrapper toolkit. The genome tree was custom plotted and annotated using the D3 JavaScript library (version 4, d3js.org).

### Par cluster gene tree

The partition gene cluster includes members of paralogous families PFam57/62, PFam50, PFam32, and PFam49 [30, 51]. Members of these gene families in newly sequenced genomes were identified based on clusters generated by CD-HIT. Protein sequences of partition gene clusters from 34 *Borreliella* and 4 relapsing-fever *Borrelia* genome sequences were downloaded from BorreliaBase [61]. Orthologous sequences were aligned with MUSCLE [131, 132] and concatenated with BIOALN. Gene trees were inferred using FastTree, mi-point rooted and low-support branches removed by using BIOTREE, and custom plotted and annotated using the D3 JavaScript library (above).

## Additional file

Additional file 1: **Figure S1.** *Borreliella* plasmid PFam32 protein neighbor-joining tree. **Figure S2.** Two examples of *Borreliella* linear plasmids with low protein coding potential. **Figure S3.** Comparative maps of linear plasmids in *Borreliella* isolates. Maps of the following linear plasmids are shown in the following panels: A, lp5; B, lp17; C, lp25; D, lp28–2, lp28–6, lp28–7 and lp28–9; E, lp28–3; F, VS116 lp28–3; G, lp28–4; H, lp28–8; I, lp32; J, lp36; K, lp38; L, lp56. **Figure S4.** The PFam54 gene cluster of the *Borreliella* lp54 plasmids. **Figure S5.** Ends of the *Borreliella* linear chromosome sequences. **Figure S6.** Comparative maps of cp9 plasmids in *Borreliella* isolates. **Figure S7.** Orphan cp32-like contigs in the *B. spielmanii* A14S genome. **Figure S8.** Rearrangements in cp32-like plasmids in NBu-*Borreliella* genomes. **Figure S9.** *B. bissettiae* DN127 66 kbp circular plasmid cp32-quad. **Figure S10.** *B. finlandensis* SV1 integration of cp32 into lp54. **Figure S11.** *Borreliella* and relapsing fever *Borrelia* PFam32 neighbor-joining tree. **Table S1.** *Borreliella* and relapsing fever *Borrelia* PFam32. (PDF 6030 kb)

## Abbreviations

ORF: open reading frame
PFam: *Borreliella* paralogous protein family
SNP: single nucleotide polymorphism
