# Supplementary material for: Primordial origin and diversification of plasmids in Lyme disease agent bacteria
Source: BMC Genomics. 2018 Mar 27;19:218. doi: 10.1186/s12864-018-4597-x (PMC5870499; doi:10.1186/s12864-018-4597-x)
Supplement: Supplementary file 1 — Figure S1. Borreliella plasmid PFam32 protein neighbor-joining tree. Figure S2. Two examples of Borreliella linear plasmids with low protein coding potential. Figure S3. Comparative maps of linear plasmids in Borreliella isolates. Maps of the following linear plasmids are shown in the following panels: A, lp5; B, lp17; C, lp25; D, lp28–2, lp28–6, lp28–7 and lp28–9; E, lp28–3; F, VS116 lp28–3; G, lp28–4; H, lp28–8; I, lp32; J, lp36; K, lp38; L, lp56. Figure S4. The PFam54 gene cluster of the Borreliella lp54 plasmids. Figure S5. Ends of the Borreliella linear chromosome sequences. Figure S6. Comparative maps of cp9 plasmids in Borreliella isolates. Figure S7. Orphan cp32-like contigs in the B. spielmanii A14S genome. Figure S8. Rearrangements in cp32-like plasmids in NBu-Borreliella genomes. Figure S9. B. bissettiae DN127 66 kbp circular plasmid cp32-quad. Figure S10. B. finlandensis SV1 integration of cp32 into lp54. Figure S11. Borreliella and relapsing fever Borrelia PFam32 neighbor-joining tree. Table S1. Borreliella and relapsing fever Borrelia PFam32. (PDF 6030 kb) [file 12864_2018_4597_MOESM1_ESM.pdf]

Supplementary Material Table of Contents

Figure legends

Figure S1. *Borreliella* plasmid PFam32 protein neighbor-joining tree 2
Figure S2. Two examples of *Borreliella* linear plasmids with low protein coding potential 2
Figure S3. Comparative maps of linear plasmids in Lyme agent *Borreliella* isolates 3
Figure S4. The PFam54 gene cluster of the *Borreliella* lp54 plasmids 4
Figure S5. Ends of the *Borreliella* linear chromosome sequences 4
Figure S6. Comparative maps of cp9 plasmids in Lyme agent *Borreliella* isolates 5
Figure S7. Orphan cp32-like contigs in the *B. spielmanii* A14S genome 5
Figure S8. Rearrangements in cp32-like plasmids in NBu-*Borreliella* genomes 6
Figure S9. *B. bissettiae* DN127 66 kbp circular plasmid cp32-quad 6
Figure S10. *B. finlandensis* SV1 integration of cp32 into lp54 6
Figure S11. *Borreliella* and relapsing fever *Borrelia* PFam32 protein neighbor-joining tree... 7

Tables

Table S1. Lyme agent *Borreliella* sequence accession numbers 8-9

References 10-11

Figures

Figure S1 12
Figure S2 13
Figure S3 A-L 14-25
Figure S4 26
Figure S5A-B 27-28
Figure S6 29
Figure S7 30
Figure S8 31
Figure S9 32
Figure S10A-B 33
Figure S11 34

Figure S1. *Borreliella* plasmid PFam32 protein neighbor-joining tree.

PFam32 amino acid sequences were aligned and an unrooted neighbor-joining tree was constructed by Clustal X (Larkin *et al*., 2007) showing the different PFam32 branches highlighed with different colors; bootstrap values from 1000 trials are shown above the branches and branches with bootstrap values below 900 are collapsed to multi-branch points. A fractional distance scale bar is shown at the lower left. All currently known PFam32 protein types from the *Borreliella* species are shown; in some types only several representative *B. burgdorferi* were included to simplify the tree. Plasmid names are indicated at the right of each branch in large text, and *Borreliella* isolates carrying them are indicated in smaller text at the branch tips. The asterisks (*) note the unusual second PFam32 protein encoded on strain B31 plasmid lp28-1 and the PFam32 genes present in small contigs of draft *B. afzelii* PKo and *B. japonica* HO14 genomes sequences (see text of article); the larger stars (★) mark the DN127 lp56 and Bol26 lp28-9 plasmids whose phylogenetic positions are inconsistent with the genome tree in figure 5 of the text. Lepto_ParA denotes the chromosomally encoded ParA protein from *Leptospira interrogans* strain UT126, a species in another spirochete genus.

Figure S2. Two examples of *Borreliella* linear plasmids with low protein coding potential.

Plasmid lp28-4 from strain PKo and plasmid lp36 from strain A14S are shown as reading frame maps are shown with the six possible reading frames (top three rightward frames and bottom three leftward); stop codons are indicated by vertical lines that span the frame rectangle, and potential start codons are indicated by short vertical lines. ORFs that appear to be intact are green, apparent pseudogenes are red, and small ORFs called by our annotation pipeline or by manual observation that may or may not be functional genes are yellow. The maps were created with DNA Strider (Douglas, 1994) and colored with Adobe ILLUSTRATOR. Paralogous protein family (PFam) numbers are given above where asterisks (*) mark the pseudogenes. Locus_tags (*e.g.*, BB_K19) identify homologous proteins that do not belong to a PFam.

Figure S3. Comparative maps of linear plasmids in *Borreliella* isolates.

Linear plasmid reading frame maps are shown with the six possible reading frames (top three are rightward frames and bottom three leftward) with stop codons indicated by vertical lines that span the frame rectangle, and potential start codons are indicated by short vertical lines (created with DNA Strider; Douglas, 1994). Green shading between maps indicates most of the homologous regions among adjacent plasmids. In each panel, the same shading color on the maps indicates regions of similar sequence. In appropriate cases, representative *B. burgdorferi* plasmids are included for comparison.

Maps of the following linear plasmids are shown in the figure panels: **A**, lp5; **B**, lp17; **C**, lp25; **D**, lp28-2, lp28-7 and lp28-9; **E**, lp28-3; **F**, strain VS116 lp28-3; **G**, lp28-4; **H**, lp28-8; **I**, lp32s; **J**, lp36; **K**, lp38; **L**, lp56.

Plasmid names are shown at the top of each panel and strain names at the left. Plasmid subtype Roman numeral names are indicated at the right in black with the species name in red text. Above the maps selected PFam numbers or names of homologous genes are shown (Paralogous Protein Families defined by Casjens *et al.* (2000, 2012); note that original related PFams 62 and 57 are merged into one PFam57. Asterisks (*) indicate obvious truncated or frame-disrupted pseudogenes, and hash marks (#) indicate draft sequences.

A "U" above an ORF marks a NBu-*Borreliella* protein that has no homologues on the *B. burgdorferi* linear plasmids. These are typified by the following examples:

U1 *B. afzelii* isolate BO23 lp17 locus_tag BLA32_05550 - unique in sequenced plasmids

U2 *B. afzelii* isolate PKo lp17 locus_tag BafPKo_D0023 and homologues

U3 *B. afzelii* isolate ACA-1 lp17 locus_tag BafACA1_D03 and homologues; unannotated pseudogene "homologue" present in *B. burgdorferi* lp17.

U4 *B. garinii* isolate PBr lp25 locus_tag BGAPBR_E0006 and homologues

U5 *B. spielmanii* isolate A14S lp28-8 locus_tag BSPA14S_N0008 and homologues

U6 *B. spielmanii* isolate A14S lp36 locus_tag BSPA14S_K0035 and homologues

U7 *B. afzelii* isolate PKo lp28-8 locus_tag BafPKo_AC0001; homologues of unknown function found adjacent to *sagE* in other species (Molloy *et al.*, 2015) and so sometimes called *sagF.*

U8 *B. afzelii* isolate PKo lp32-10 locus_tag BafPKo_Q0008 - unique in sequenced plasmids

U9 *B. afzelii* isolate PKo lp38 BafPKo_J0009 and homologues

Figure S4. The PFam54 gene cluster of the *Borreliella* lp54 plasmids.

The cluster of PFam54 genes that all lp54 plasmids carry a near their right ends is shown for all the *Borreliella* isolates for which they have been sequenced. Individual genes are depicted as bars whose pointed ends indicate the direction of transcription. The cluster is bounded by black vertical lines in the figure, and blue vertical lines bound the central much more variable region. Individual isolates are indicated on the left, and species with lp54 subtype in Roman numerals is indicated on the right. All the PFam54 genes are related to some degree, and in the variable region genes of the same color form groups that are ≥65% identical in amino acid sequence. A few outliers just outside that limit are indicated above the gene with the percent identity to the rest of the group. The rightmost identifying portion of their GenBank locus-tags are shown on each gene, asterisks (*) denote the longer pseudogenes that are truncated or have reading frame disruptions, and the red triangle in the *B. finlandensis* line indicates the site of cp32 integration. Spaces between genes in the variable region do not indicate the presence of DNA, but only serve to allow better vertical alignment of the different gene types indicated by the different colors. Black X's mark regions that have not yet been sequenced.

Figure S5. Ends of the *Borreliella* linear chromosome sequences.

Maps of the sizeable ORFs at the left and right end regions of the linear chromosomes of the available NBu-*Borreliella* sequences are shown in parts **A** and **B**, respectively. Two *B. burgdorferi* termini are shown for comparison. Predicted genes are shown as rectangles with pointed ends that indicate the direction of transcription and the B31 gene names are indicated on the B31 maps.

Heavy black horizontal lines mark the extent of the terminal sequence. Green genes are in terminal extensions relative to the common short chromosomes; the paralogous protein family (PFam abbreviated here as PF) is given above each such gene and small asterisks (*) mark pseudogenes. Numbers above each map give the bp lengths of common chromosomal genes (including the stop codon), distance between genes (a negative value for the latter indicates a postulated gene overlap), and distance from the most terminal common gene (*bb_001* at left end except for the *B. valaisiana* strains where it is *bb_002*; *bb_843* at right end) to the end of the sequence. Parentheses mark the inversion in Tom4006 relative to VS116. Scales in kbp are shown above that begin at the start of the indicated gene.

We note that because the linear *Borreliella* replicons have closed hairpin telomeres, their terminal fragments are not ligated into plasmid DNA libraries. Thus, sequences determined by dideoxy-sequencing of such libraries do not include the near terminal sequences. Sequences that include the telomere are marked with a large asterisk (*); black asterisks indicate telomeres that were purposefully sequenced, and gray asterisks indicate sequences that appear include at least most of the ~25 bp telomere sequence. Jagged termini of genes mark locations where such early sequencing methods did not reach the end of the terminal gene.

Figure S6. Comparative maps of cp9 plasmids in *Borreliella* isolates.

Aligned open reading frame maps were created as described for figure S3. Asterisks (*) indicate the accession numbers for two putative A14S contigs that were not "closed". They are very likely to be cp9 contigs because of their unique high similarity to the cp9s of other *Borreliella* species.

Figure S7. Orphan cp32-like contigs in the *B. spielmanii* A14S genome.

Strain A14S nucleotide sequence contigs are shown as horizontal bars that are aligned with homologous sequences in the strain B31 cp32-1 plasmid. The cp32-1 plasmid is circular (opened for linear display here at an arbitrary point as in accession number AE001575), and its six frame open reading frame map (created as described for figure S3 by DNA Strider; Douglas, 1994) is shown above.

Homologies for two contigs, ABKB02000023 and ABKB02000034 cross the location at which the map was opened; however, this homology is only indicated at one end of the map. The location of the cp32-1 PFam32 gene (see article text) is indicated in green on the map and contig bars that encode whole or parts of PFam32 gene are colored green.

Figure S8. Rearrangements in cp32-like plasmids in non-*burgdorferi* *Borreliella* genomes.

An ORF map (see figure S3) of strain B31 cp32-1 is shown above for comparison, and the NBu-*Borreliella* cp32s with long rearrangements are indicated by magenta bars below; long deletions are indicated by thin magenta lines and insertions and replacements by thick blue bars. The variable regions shown above the map were discussed and defined by Casjens *et al.* (2012). The ancient triplication that created PFam148 (marked in purple above; see text) includes strain B31 cp32-1 genes *bb_p03*, *bb_p04* and *bb_p05.*

Figure S9. *B. bissettiae* DN127 66 kbp circular plasmid cp32-quad.

ORF maps (see figure S3) of strain DN127 cp32-quad and cp32-7 are shown along top and right axes for orientation. The indicated section of the cp32-quad sequence was manually inverted so that all the internal sequence similarities could be displayed in one plot. The dot plot was created by DNA Strider (Douglas, 1994) with a scan window of 13 identities in 15 bp. The location of PFam32 protein encoding genes are indicated above.

Figure S10. *B. finlandensis* SV1 integration of cp32 into lp54.

A. A dot plot that compares strain B31 lp54 and SV1 lp54 is shown that was created by DNA Strider (Douglas, 1994) with a scan window of 17 identities in 23 bp. Similar comparison of SV1 lp54 to a cp32 plasmid showed the location of the cp32-11 sequence in this lp54 (indicated by the yellow bar). The B31 lp54 ORFs are indicated below the plot.

B. The putative sequences of the parental lp54 and cp32 plasmids are shown. The nonhomologous crossover point that generates the SV1 fused plasmid is marked by a carat (^).

Figure S11. *Borreliella* and relapsing fever clade *Borrelia* PFam32 protein neighbor-joining tree.

The tree was constructed as in figure S1. The relapsing fever PFam32 proteins, indicated by "species_strain name_plasmid name" at their branch tips, and the branches on which they reside are colored red (note that among these plasmids only *B. recurrentis* A1 plasmid pL53 encodes two such proteins). The *Borreliella* PFam32 plasmid types are indicated in large black text at the right of the black branches. All known PFam32 protein types from the *Borreliella* species are shown; in some types only several representative *B. burgdorferi* were included to simplify the tree.

Table S1

Borreliella sequence accession numbers

Part 1. Accession numbers of anecdotal plasmid sequences listed in figure 1

Strain	*B. afzelii* BO23	*B. garinii* 20047	*B. japonica* HO14
**Plasmid**			
cp9	CP018274	–	–
cp26	CP018266	CP018750	FMTE01000007
lp17	CP018269	CP018751	–
lp25	–	–	FMTE01000009
lp28-3	CP018265	–	–
lp28-4	CP018268	–	–
lp28-7	CP018267	CP018749	–
lp28-8	CP018264	–	FMTE01000008
lp36	–	CP018746	–
lp38	CP018263	–	–
lp54	CP018263	CP018745	FMTE01000005

Part 2. Accession numbers of plasmid sequences not listed in figure 1

Plasmid	cp9(cp8.3)	cp26	lp54
**Species / Isolate**			
B. afzelii Tom3017	–	NZ_CP009213	NZ_CP009214
MMS	–	–	AJ786368 [a]
B. bavariensis PBi	–	CP000014	CP000015
BgVir	–	CP003201	CP003202
ZQ1	–	–	AJ786369[a]
B. garinii lp21	U03641	–	–
B. valaisiana Tom4006	–	NZ_CP009118	NZ_CP009119
B. chilensis VA1	–	CP009911	CP009912

Table S1 (cont.)

Part 3. Accession numbers of chromosomes

Species / Isolate	Chromosome	Reference
B. afzelii Tom3017	NZ_CP009212	(Kurilshikov *et al*., 2014)
HLJ01	CP003883	(Jiang *et al*., 2012b)
R-IP3	AF008219	(Casjens *et al*., 1997)
B. bavariensis PBi	CP000013	(Glöckner *et al*., 2004)
BgVir	CP003151	(Brenner *et al*., 2012)
SZ	CP007564	(Wu *et al*., 2014)
NMJW1	CP003866	(Jiang *et al*., 2012a)
B. burgdorferi B31	AE000783	(Fraser *et al*., 1997)
Sh-2-82	AF008218	(Casjens *et al*., 1997)
B. valaisiana Tom4006	NZ_CP009117	(Kurilshikov *et al*., 2014)
B. chilensis VA1	CP009910	(Huang *et al*., 2015)

Footnote

a. Only the PFam54 cluster sequence is known; see figure S4

Supplementary Material References

Brenner, E. V., Kurilshikov, A. M., Stronin, O. V., Fomenko, N. V., 2012. Whole-genome sequencing of *Borrelia garinii* BgVir, isolated from Taiga ticks (*Ixodes persulcatus*). J Bacteriol 194, 5713.

Casjens, S., Murphy, M., DeLange, M., Sampson, L., van Vugt, R., Huang, W. M., 1997. Telomeres of the linear chromosomes of Lyme disease spirochaetes: nucleotide sequence and possible exchange with linear plasmid telomeres. Mol. Microbiol. 26, 581-96.

Casjens, S., Palmer, N., van Vugt, R., Huang, W. M., Stevenson, B., Rosa, P., Lathigra, R., Sutton, G., Peterson, J., Dodson, R. J., Haft, D., Hickey, E., Gwinn, M., White, O., Fraser, C. M., 2000. A bacterial genome in flux: the twelve linear and nine circular extrachromosomal DNAs in an infectious isolate of the Lyme disease spirochete *Borrelia burgdorferi*. Mol Microbiol 35, 490-516.

Casjens, S. R., Mongodin, E. F., Qiu, W. G., Luft, B. J., Schutzer, S. E., Gilcrease, E. B., Huang, W. M., Vujadinovic, M., Aron, J. K., Vargas, L. C., Freeman, S., Radune, D., Weidman, J. F., Dimitrov, G. I., Khouri, H. M., Sosa, J. E., Halpin, R. A., Dunn, J. J., Fraser, C. M., 2012. Genome stability of Lyme disease spirochetes: comparative genomics of *Borrelia burgdorferi* plasmids. PLoS One 7, e33280.

Douglas, S. E., 1994. DNA Strider. A Macintosh program for handling protein and nucleic acid sequences. Methods Mol. Biol. 25, 181-94.

Fraser, C. M., Casjens, S., Huang, W. M., Sutton, G. G., Clayton, R., Lathigra, R., White, O., Ketchum, K. A., Dodson, R., Hickey, E. K., Gwinn, M., Dougherty, B., Tomb, J. F., Fleischmann, R. D., Richardson, D., Peterson, J., Kerlavage, A. R., Quackenbush, J., Salzberg, S., Hanson, M., van Vugt, R., Palmer, N., Adams, M. D., Gocayne, J., Venter, J. C., 1997. Genomic sequence of a Lyme disease spirochaete, *Borrelia burgdorferi*. Nature 390, 580-6.

Glöckner, G., Lehmann, R., Romualdi, A., Pradella, S., Schulte-Spechtel, U., Schilhabel, M., Wilske, B., Suhnel, J., Platzer, M., 2004. Comparative analysis of the *Borrelia garinii* genome. Nucleic Acids Res 32, 6038-46.

Huang, W., Ojaimi, C., Fallon, J. T., Travisany, D., Maass, A., Ivanova, L., Tomova, A., Gonzalez-Acuna, D., Godfrey, H. P., Cabello, F. C., 2015. Genome Sequence of *Borrelia chilensis* VA1, a South American Member of the Lyme Borreliosis Group. Genome Announc 3, e01535-14.

Jiang, B., Yao, H., Tong, Y., Yang, X., Huang, Y., Jiang, J., Cao, W., 2012a. Genome sequence of *Borrelia garinii* strain NMJW1, isolated from China. J Bacteriol 194, 6660-1.

Jiang, B. G., Zheng, Y. C., Tong, Y. G., Jia, N., Huo, Q. B., Fan, H., Ni, X. B., Ma, L., Yang, X. F., Jiang, J. F., Cao, W. C., 2012b. Genome sequence of *Borrelia afzelii* Strain HLJ01, isolated from a patient in China. J Bacteriol 194, 7014-5.

Kurilshikov, A. M., Fomenko, N. V., Stronin, O. V., Tikunov, A. Y., Kabilov, M. R., Tupikin, A. E., Tikunova, N. V., 2014. Complete Genome Sequencing of *Borrelia valaisiana* and *Borrelia afzelii* Isolated from *Ixodes persulcatus* Ticks in Western Siberia. Genome Announc 2, e01315-14.

Larkin, M. A., Blackshields, G., Brown, N. P., Chenna, R., McGettigan, P. A., McWilliam, H., Valentin, F., Wallace, I. M., Wilm, A., Lopez, R., Thompson, J. D., Gibson, T. J., Higgins, D. G., 2007. Clustal W and Clustal X version 2.0. Bioinformatics 23, 2947-8.

Molloy, E. M., Casjens, S. R., Cox, C. L., Maxson, T., Ethridge, N. A., Margos, G., Fingerle, V., Mitchell, D. A., 2015. Identification of the minimal cytolytic unit for streptolysin S and an expansion of the toxin family. BMC Microbiol 15, 141.

Wu, Q., Liu, Z., Li, Y., Guan, G., Niu, Q., Chen, Z., Luo, J., Yin, H., 2014. Genome Sequence of *Borrelia garinii* Strain SZ, Isolated in China. Genome Announc 2, e00010-14.

Figure S1

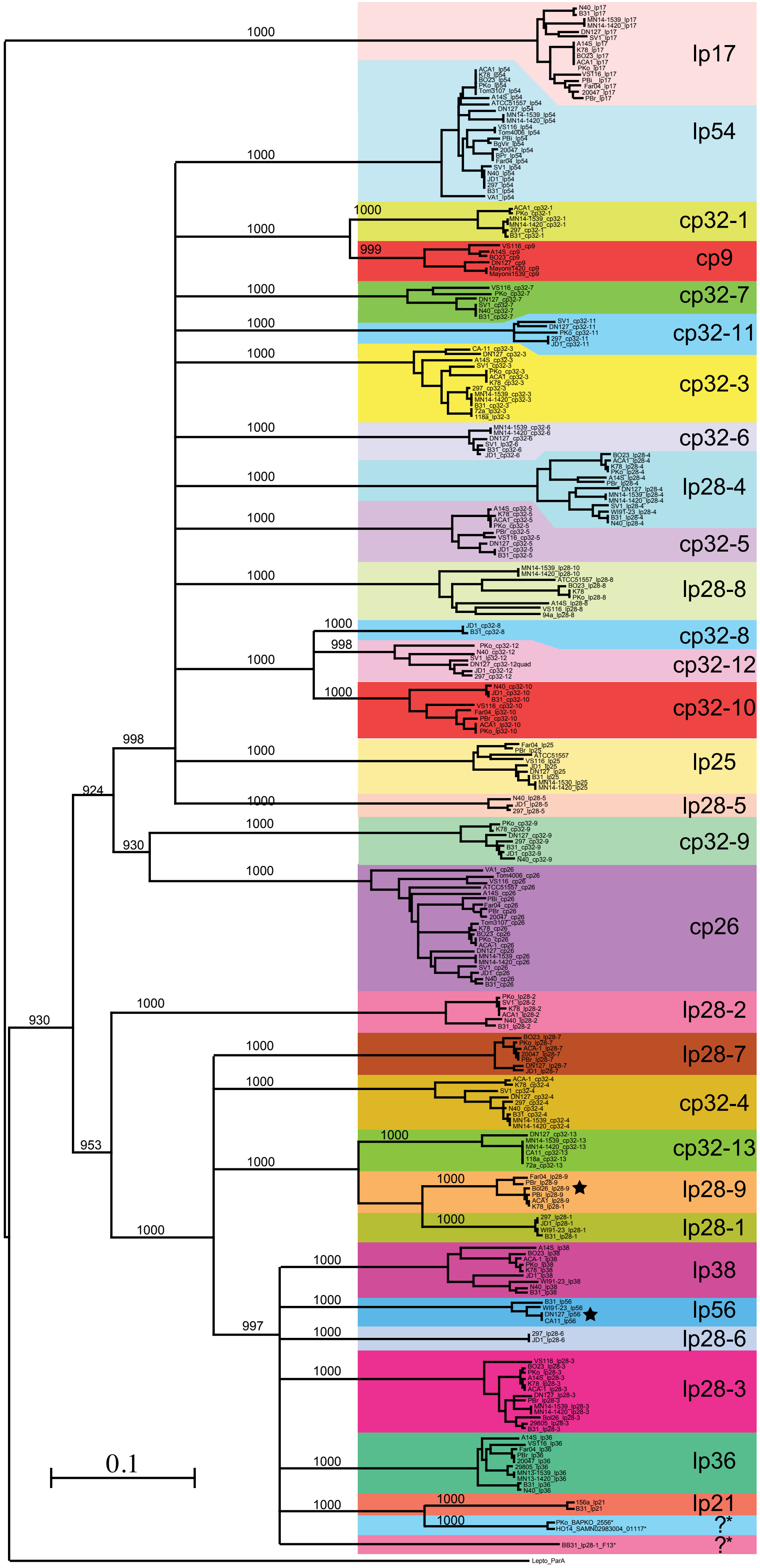
lp17
lp54
cp32-1
cp9
cp32-7
cp32-11
cp32-3
cp32-6
lp28-4
cp32-5
lp28-8
cp32-8
cp32-12
cp32-10
lp25
lp28-5
cp32-9
cp26
lp28-2
lp28-7
cp32-4
cp32-13
lp28-9
lp28-1
lp38
lp56
lp28-6
lp28-3
lp36
lp21
?*
?*
0.1

Figure S2

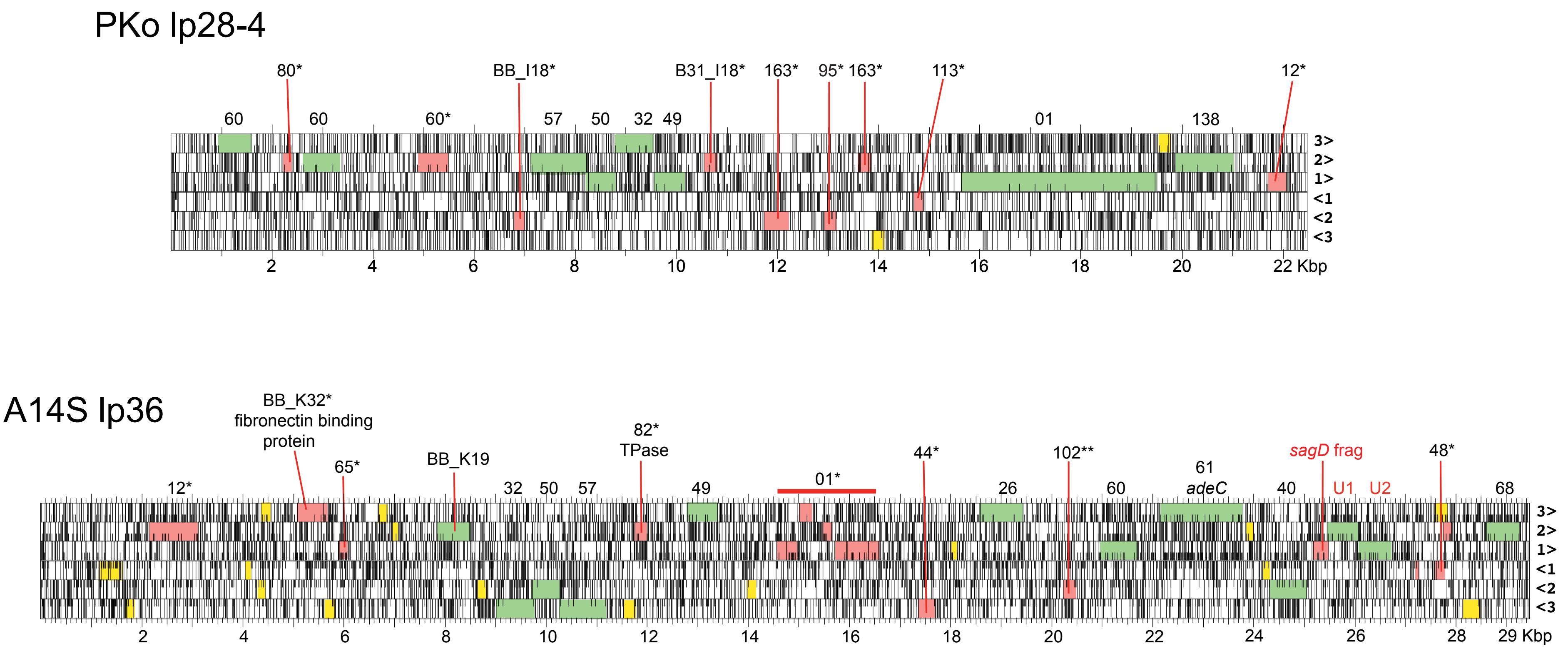
PKo lp28-4
80*
BB_I18*
B31_I18*
163*
95*
163*
113*
12*
60
60
60*
57
50
32
49
01
138
3>
2>
1>
<1
<2
<3
2
4
6
8
10
12
14
16
18
20
22 Kbp
A14S lp36
BB_K32*
fibronectin binding
protein
82*
TPase
65*
BB_K19
44*
102**
61
sagD frag
48*
01*
12*
32
50
57
49
26
60
adeC
40
U1
U2
68
3>
2>
1>
<1
<2
<3
2
4
6
8
10
12
14
16
18
20
22
24
26
28
29 Kbp

lp5

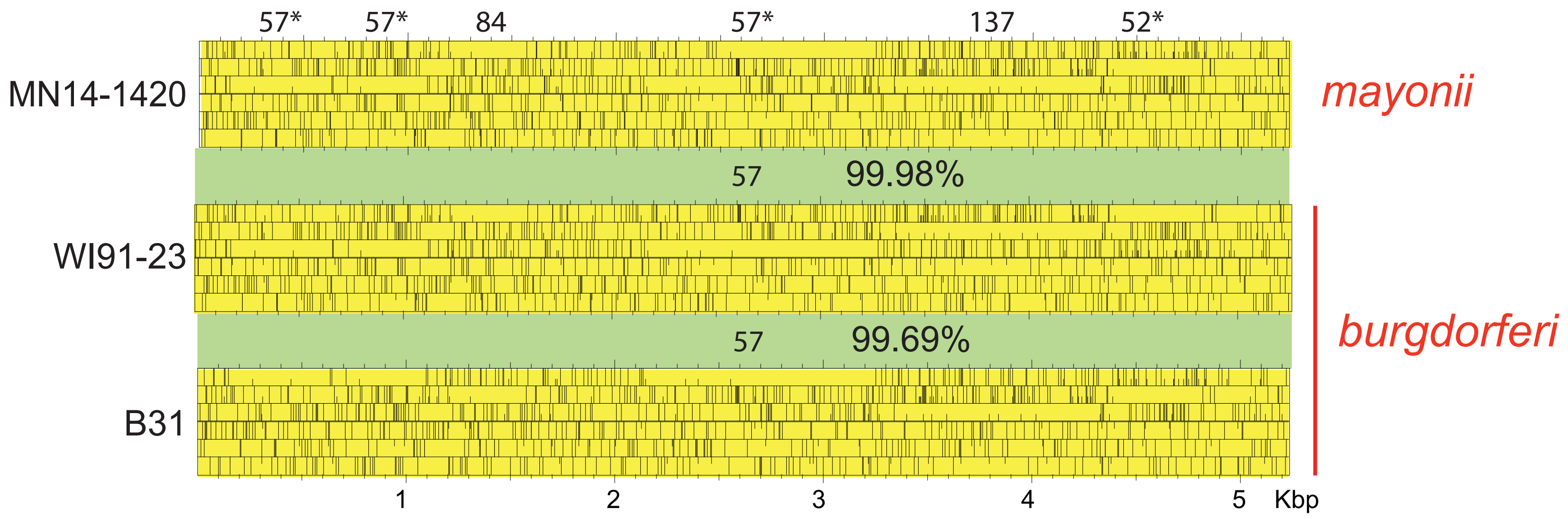
57*
57*
84
57*
137
52*
MN14-1420
mayonii
57
99.98%
WI91-23
burgdorferi
57
99.69%
B31
1
2
3
4
5
Kbp

lp17

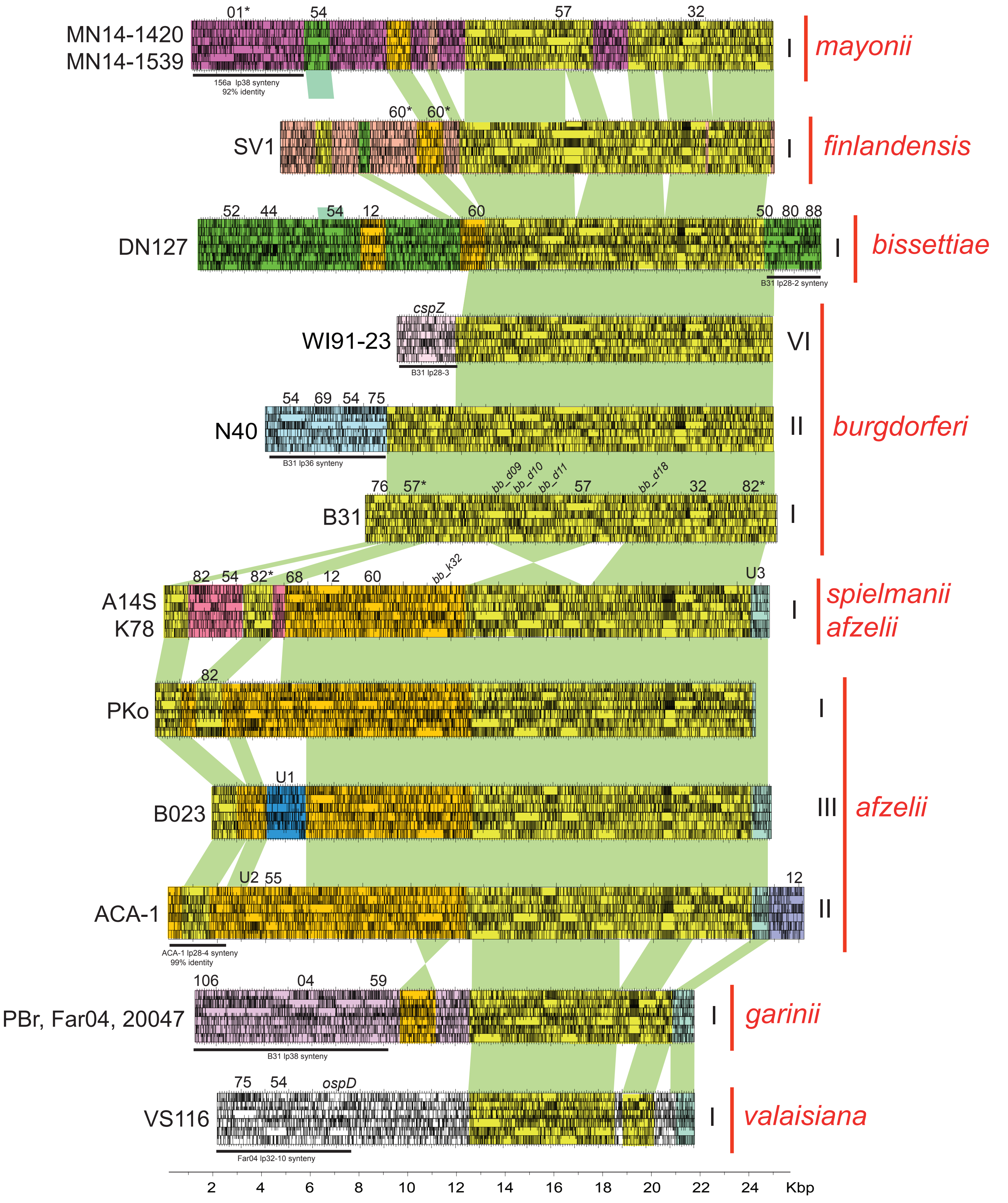
MN14-1420
MN14-1539
01*
54
57
32
156a lp38 synteny
92% identity
I
mayonii
SV1
60*
60*
I
finlandensis
DN127
52
44
54
12
60
50
80
88
B31 lp28-2 synteny
I
bissettiae
WI91-23
cspZ
B31 lp28-3
VI
N40
54
69
54
75
B31 lp36 synteny
II
burgdorferi
B31
76
57*
bb_d09
bb_d10
bb_d11
57
bb_d18
32
82*
I
A14S
K78
82
54
82*
68
12
60
bb_k32
U3
I
spielmanii
afzelii
PKo
82
I
B023
U1
III
afzelii
ACA-1
U2
55
12
ACA-1 lp28-4 synteny
99% identity
II
PBr, Far04, 20047
106
04
59
B31 lp38 synteny
I
garinii
VS116
75
54
ospD
Far04 lp32-10 synteny
I
valaisiana
2
4
6
8
10
12
14
16
18
20
22
24
Kbp

Figure S3C

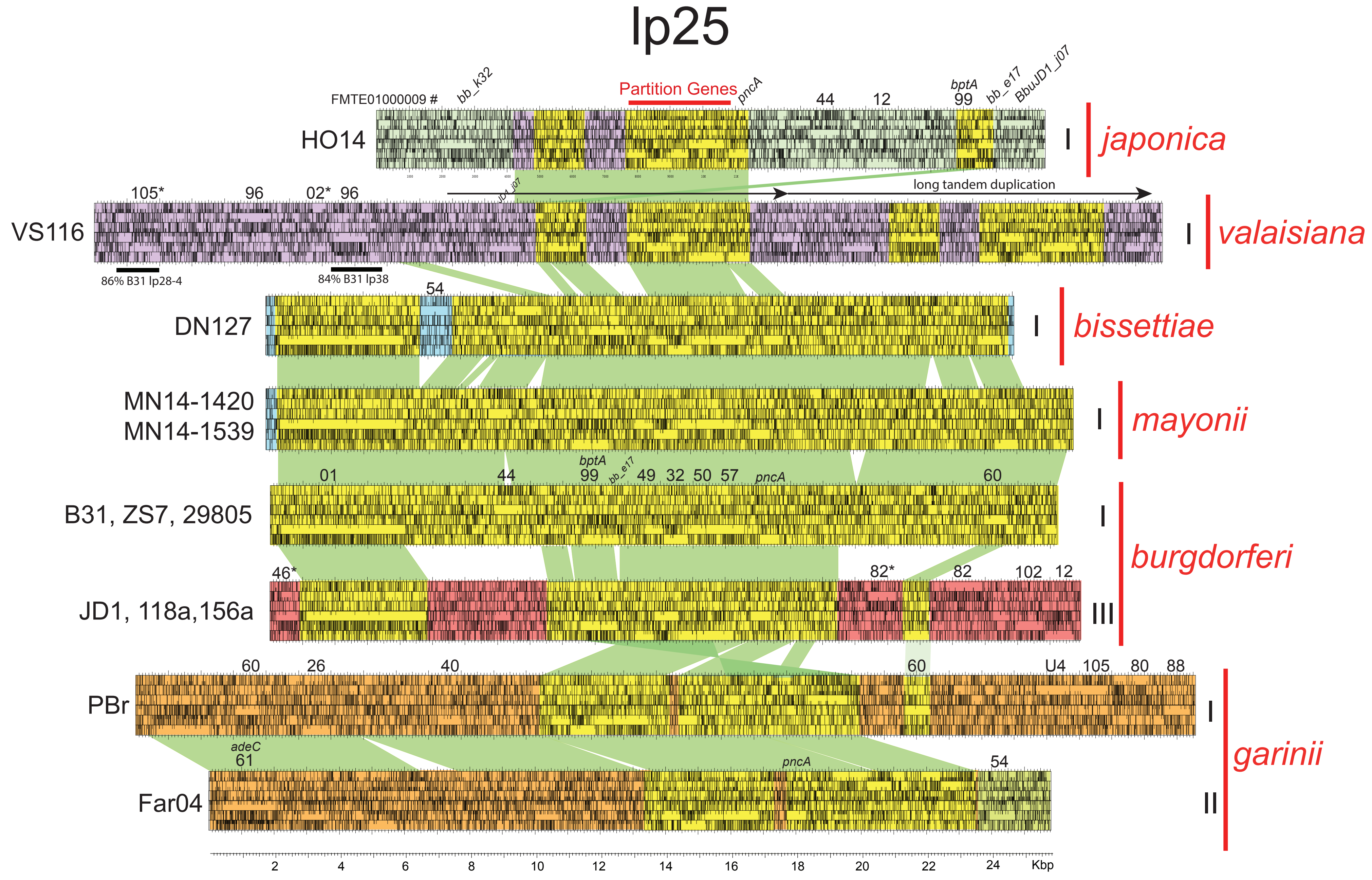
lp25
FMTE01000009 #
bb_k32
Partition Genes
pncA
44
12
bptA
99
bb_e17
BbuJD1_j07
HO14
I
japonica
long tandem duplication
105*
96
02*
96
VS116
I
valaisiana
86% B31 lp28-4
84% B31 lp38
54
DN127
I
bissettiae
MN14-1420
MN14-1539
I
mayonii
01
44
bptA
99
bb_e17
49
32
50
57
pncA
60
B31, ZS7, 29805
I
burgdorferi
46*
82*
82
102
12
JD1, 118a,156a
III
60
26
40
60
U4
105
80
88
PBr
I
garinii
adeC
61
pncA
54
Far04
II
2
4
6
8
10
12
14
16
18
20
22
24
Kbp

lp28-2, -7 & 9

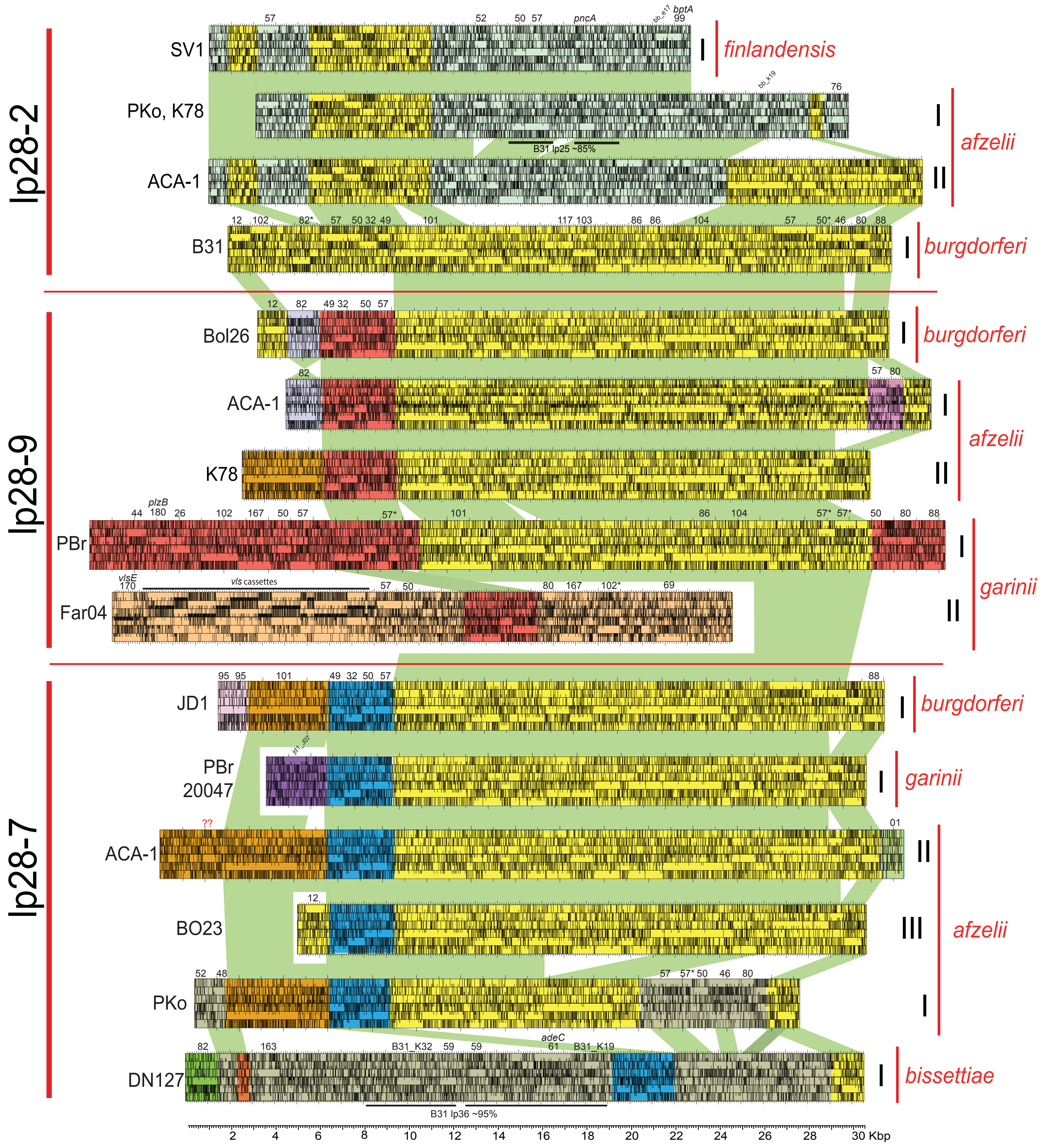

lp28-2
lp28-9
lp28-7
SV1
PKo, K78
ACA-1
B31
Bol26
ACA-1
K78
PBr
Far04
JD1
PBr 20047
ACA-1
BO23
PKo
DN127
finlandensis
afzelii
burgdorferi
burgdorferi
afzelii
garinii
burgdorferi
garinii
afzelii
bissettiae
pncA
bptA
B31 lp25 ~85%
plzB
vlsE
vls cassettes
adeC
B31_K32
B31_K19
B31 lp36 ~95%
2 4 6 8 10 12 14 16 18 20 22 24 26 28 30 Kbp

lp28-3

Figure S3E

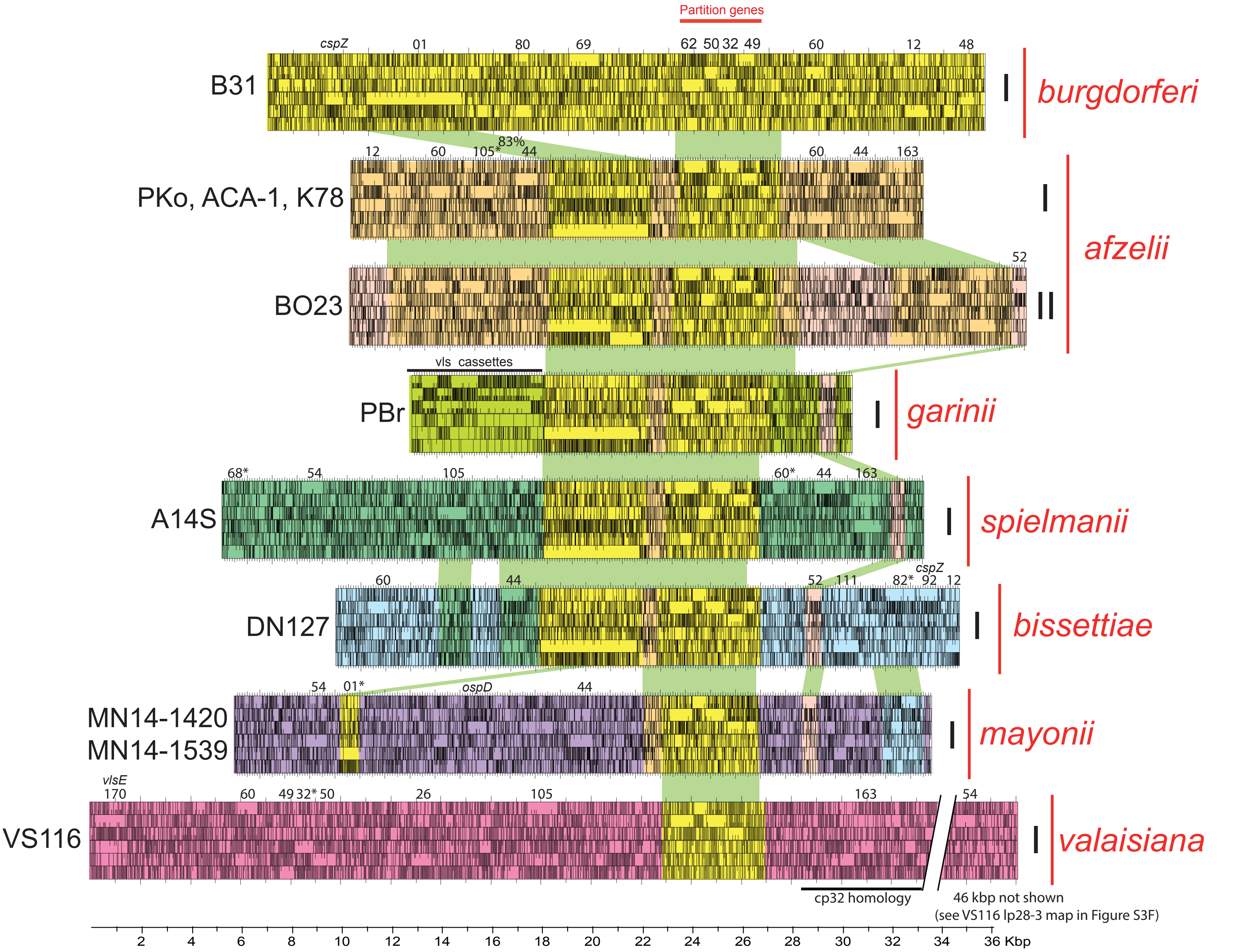

B. valaisiana VS116 lp28-3

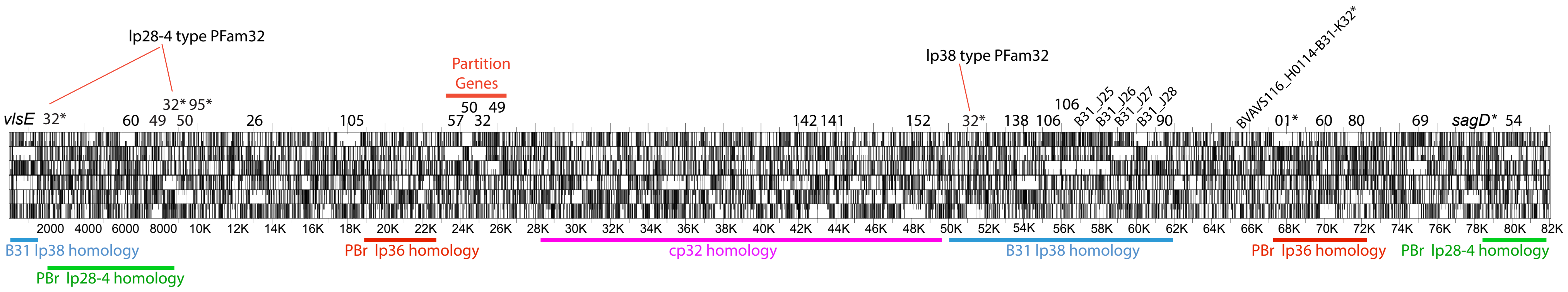

lp28-4

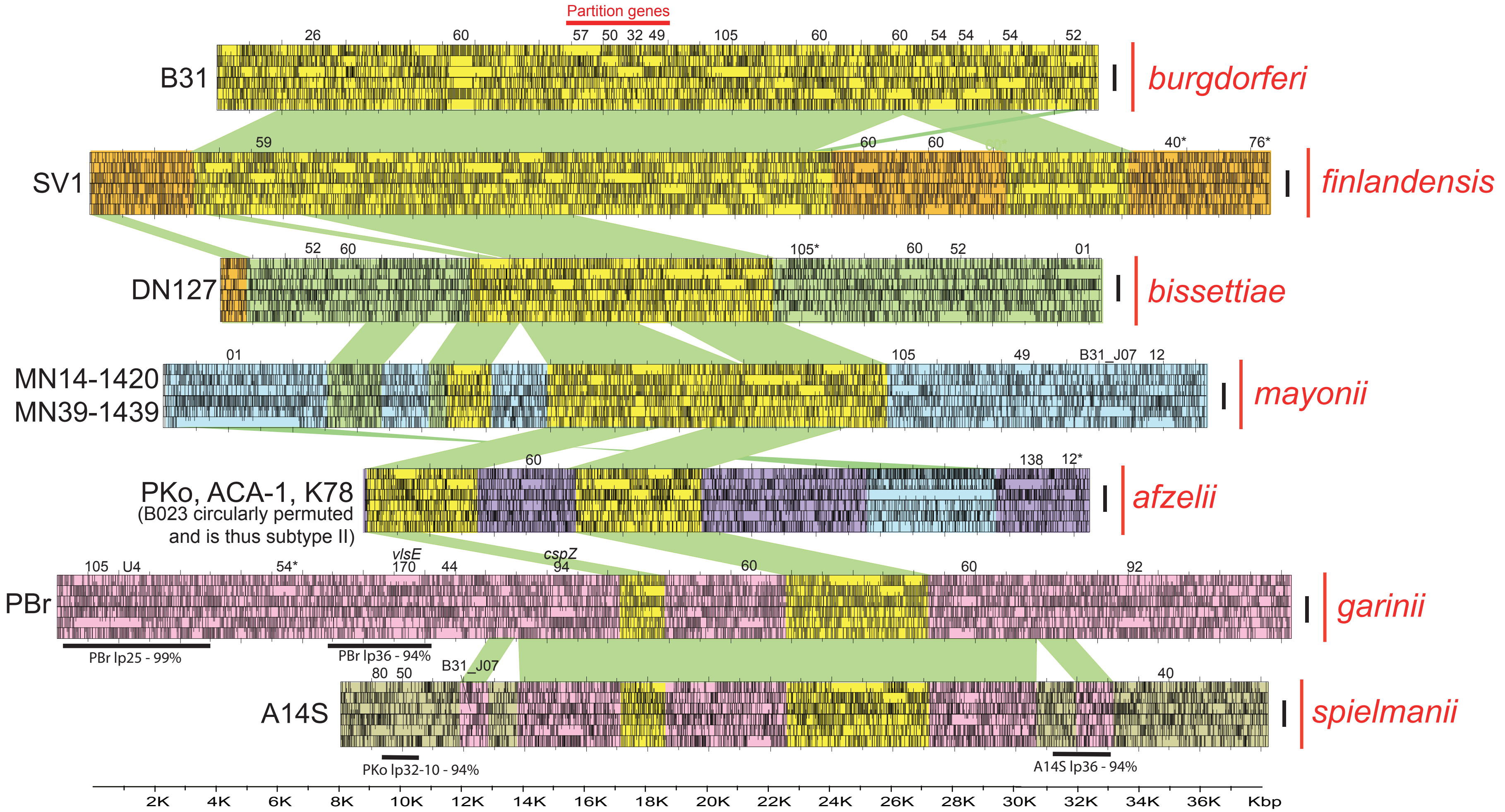
Partition genes
B31
SV1
DN127
MN14-1420
MN39-1439
PKo, ACA-1, K78
(B023 circularly permuted
and is thus subtype II)
PBr
A14S
burgdorferi
finlandensis
bissettiae
mayonii
afzelii
garinii
spielmanii
vlsE
cspZ
B31_J07
PBr lp25 - 99%
PBr lp36 - 94%
PKo lp32-10 - 94%
A14S lp36 - 94%
2K 4K 6K 8K 10K 12K 14K 16K 18K 20K 22K 24K 26K 28K 30K 32K 34K 36K Kbp

Figure S3H

lp28-8

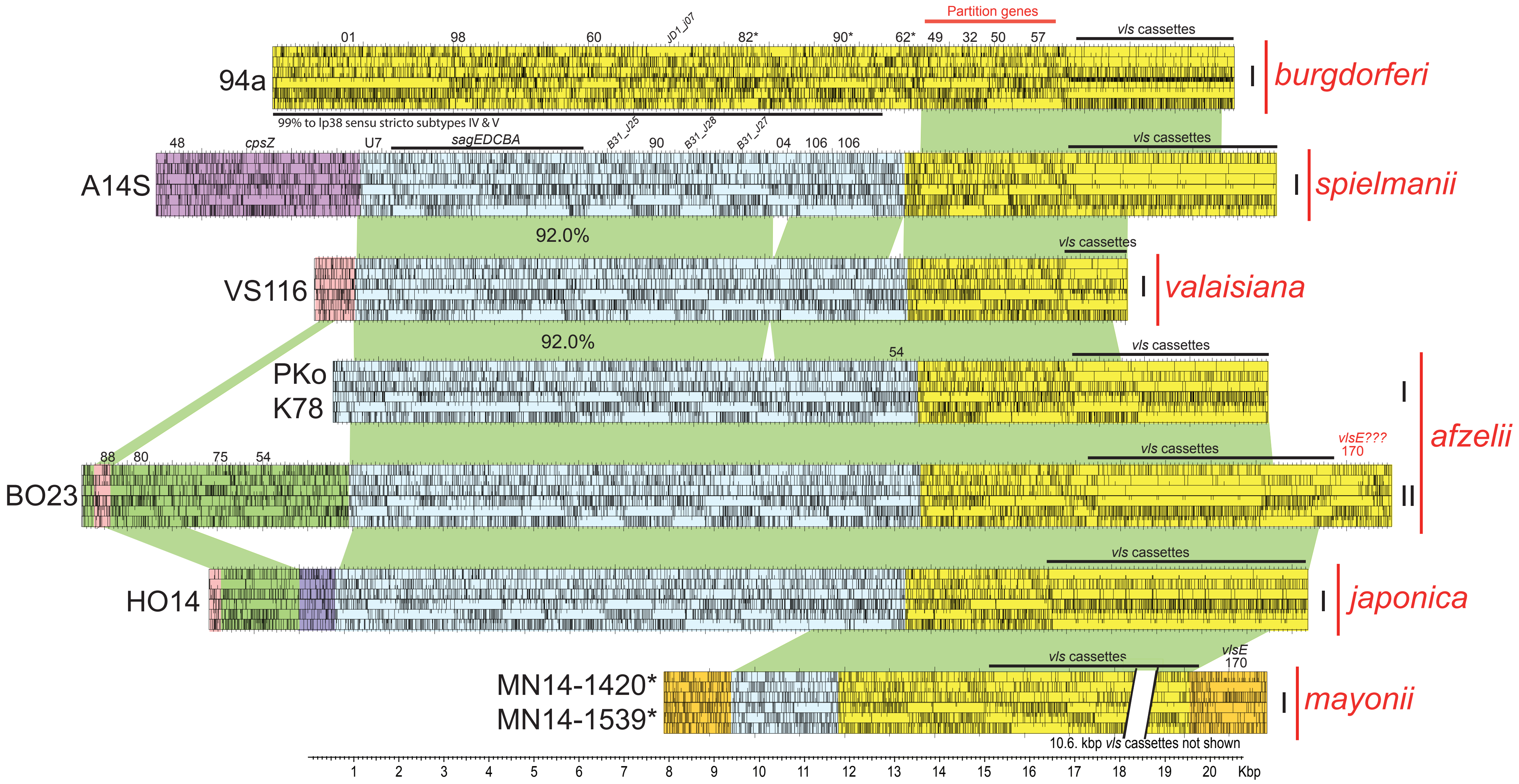

* Figure includes *B. mayonii* “lp28-10s” (see text)

Figure S3I

lp32-3, -6, -10 & -12

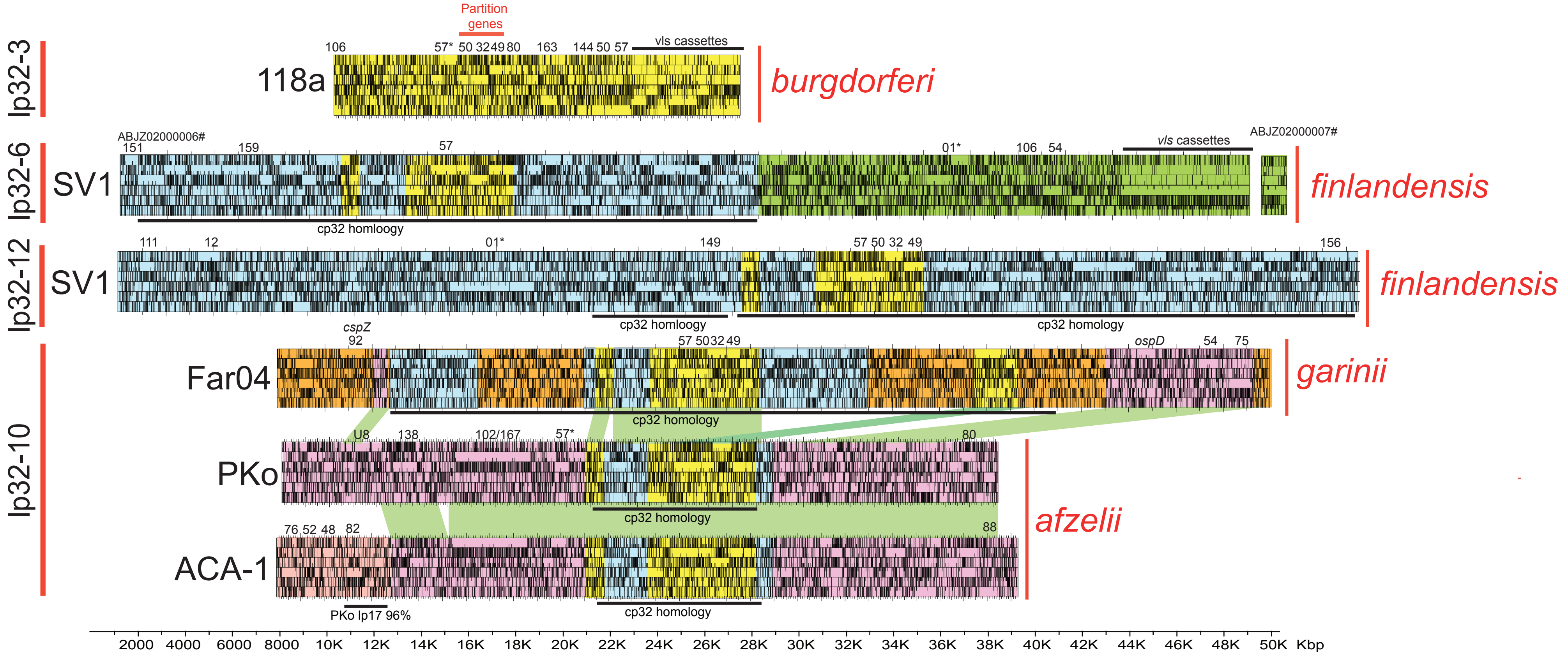

The two contigs of the SV1 plasmid lp32-6 were not “closed” but were experimentally connected to the same plasmid; the gap was not sequenced

Yellow shading marks regions that have similarity to strain 118a plasmid lp32-3. This largely marks the partition gene clusters which, although they have some similarity, encode PFam32 proteins of dfferent types in the different types of plasmid.

lp36

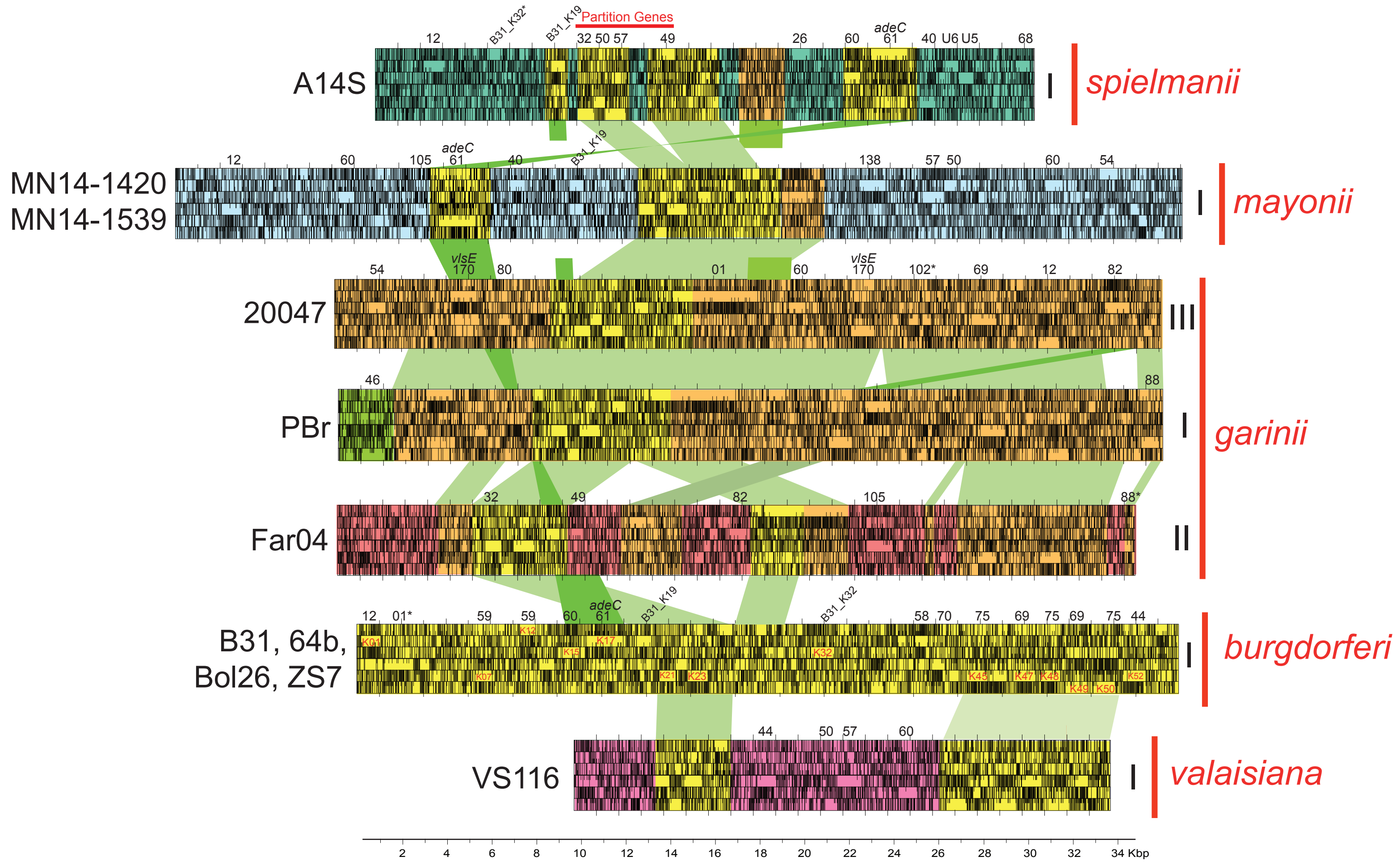
Partition Genes
adeC
A14S
MN14-1420
MN14-1539
20047
PBr
Far04
B31, 64b,
Bol26, ZS7
VS116
vlsE
I
III
II
spielmanii
mayonii
garinii
burgdorferi
valaisiana
34 Kbp

lp38

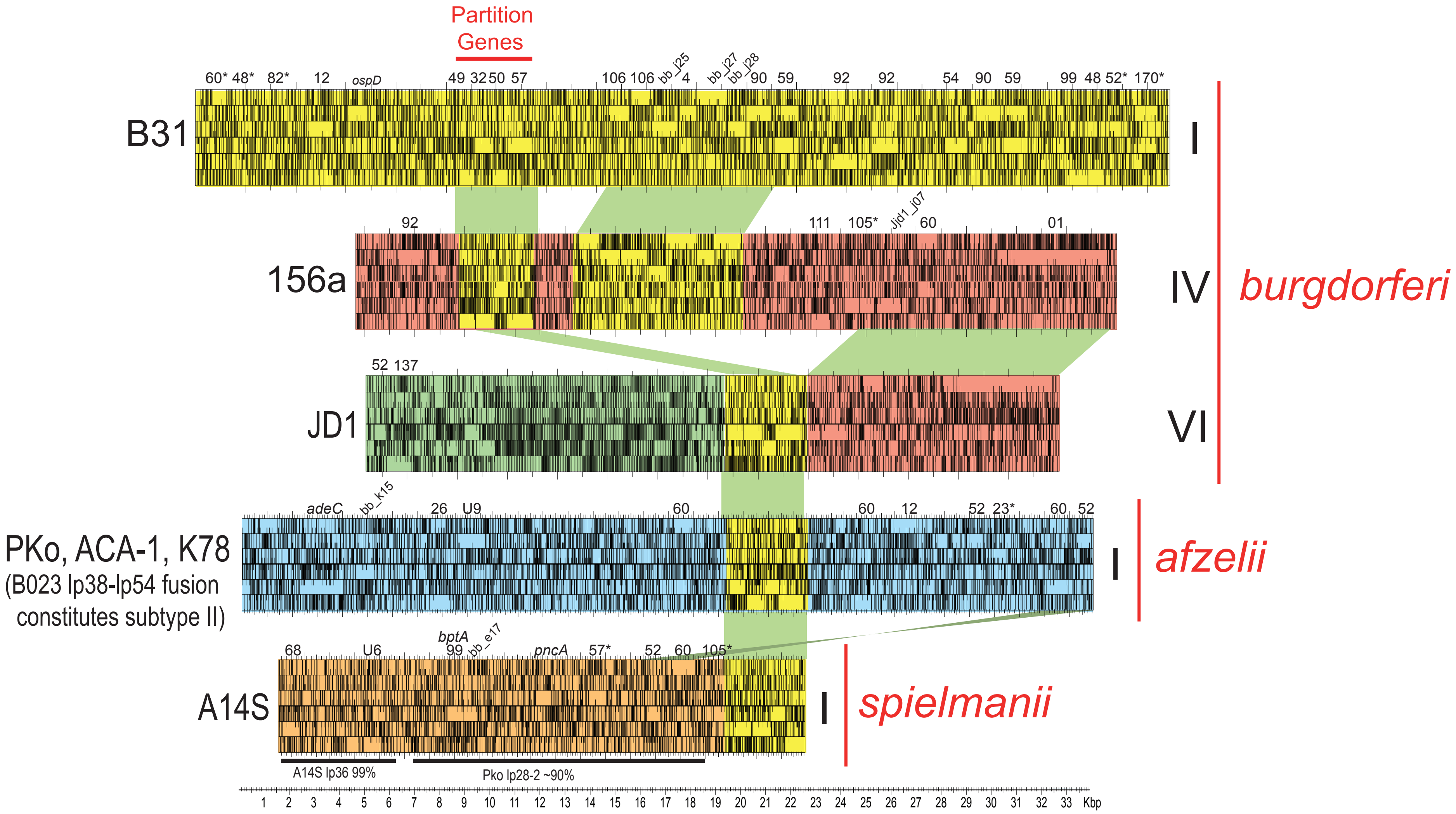
Partition Genes
60* 48* 82* 12 ospD 49 32 50 57 106 106 bb_j25 4 bb_j27 bb_j28 90 59 92 92 54 90 59 99 48 52* 170*
B31
I
92 111 105* Jid1_j07 60 01
156a
IV
burgdorferi
52 137
JD1
VI
adeC bb_k15 26 U9 60 60 12 52 23* 60 52
PKo, ACA-1, K78
(B023 lp38-lp54 fusion constitutes subtype II)
I
afzelii
68 U6 bptA 99 bb_e17 pncA 57* 52 60 105*
A14S
I
spielmanii
A14S lp36 99%
Pko lp28-2 ~90%
1 2 3 4 5 6 7 8 9 10 11 12 13 14 15 16 17 18 19 20 21 22 23 24 25 26 27 28 29 30 31 32 33 Kbp

lp56

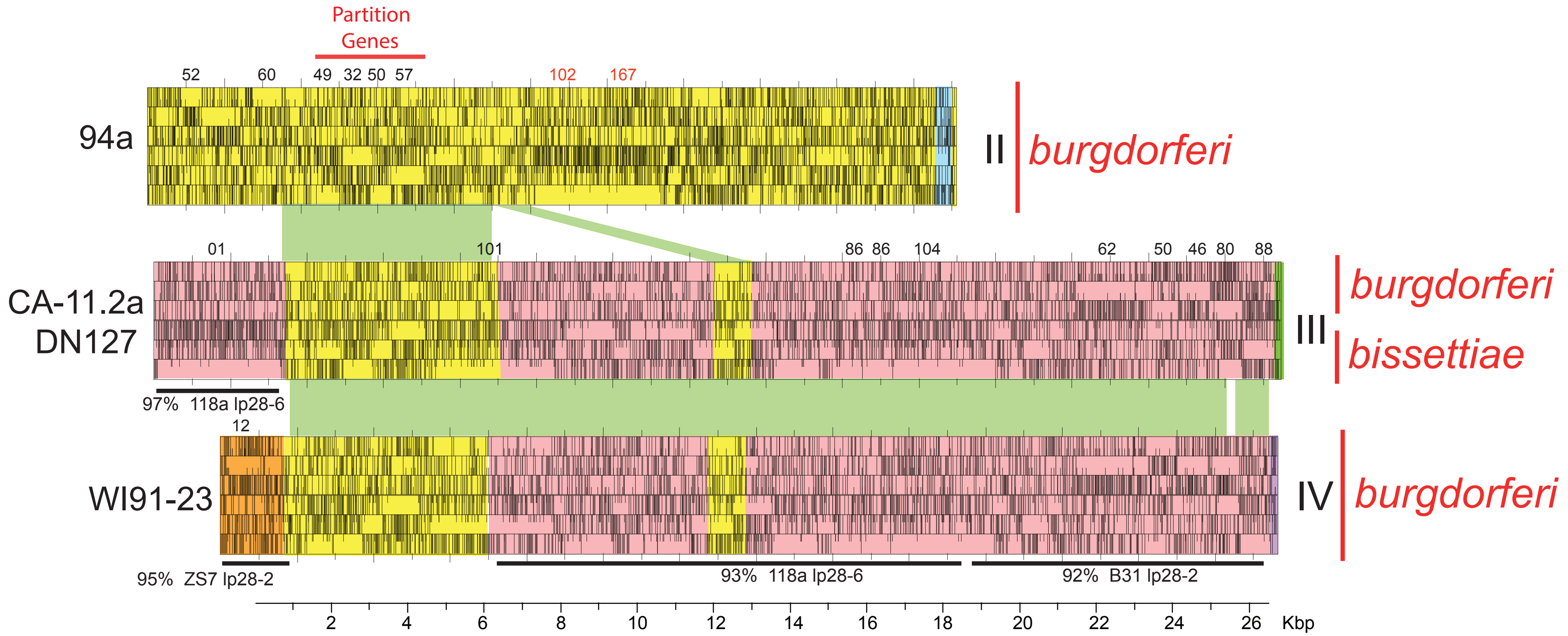
Partition
Genes
52
60
49 32 50 57
102 167
94a
II
burgdorferi
01
101
86 86 104
62 50 46 80 88
CA-11.2a
DN127
III
burgdorferi
bissettiae
97% 118a lp28-6
12
WI91-23
IV
burgdorferi
95% ZS7 lp28-2
93% 118a lp28-6
92% B31 lp28-2
2 4 6 8 10 12 14 16 18 20 22 24 26 Kbp

Figure S4

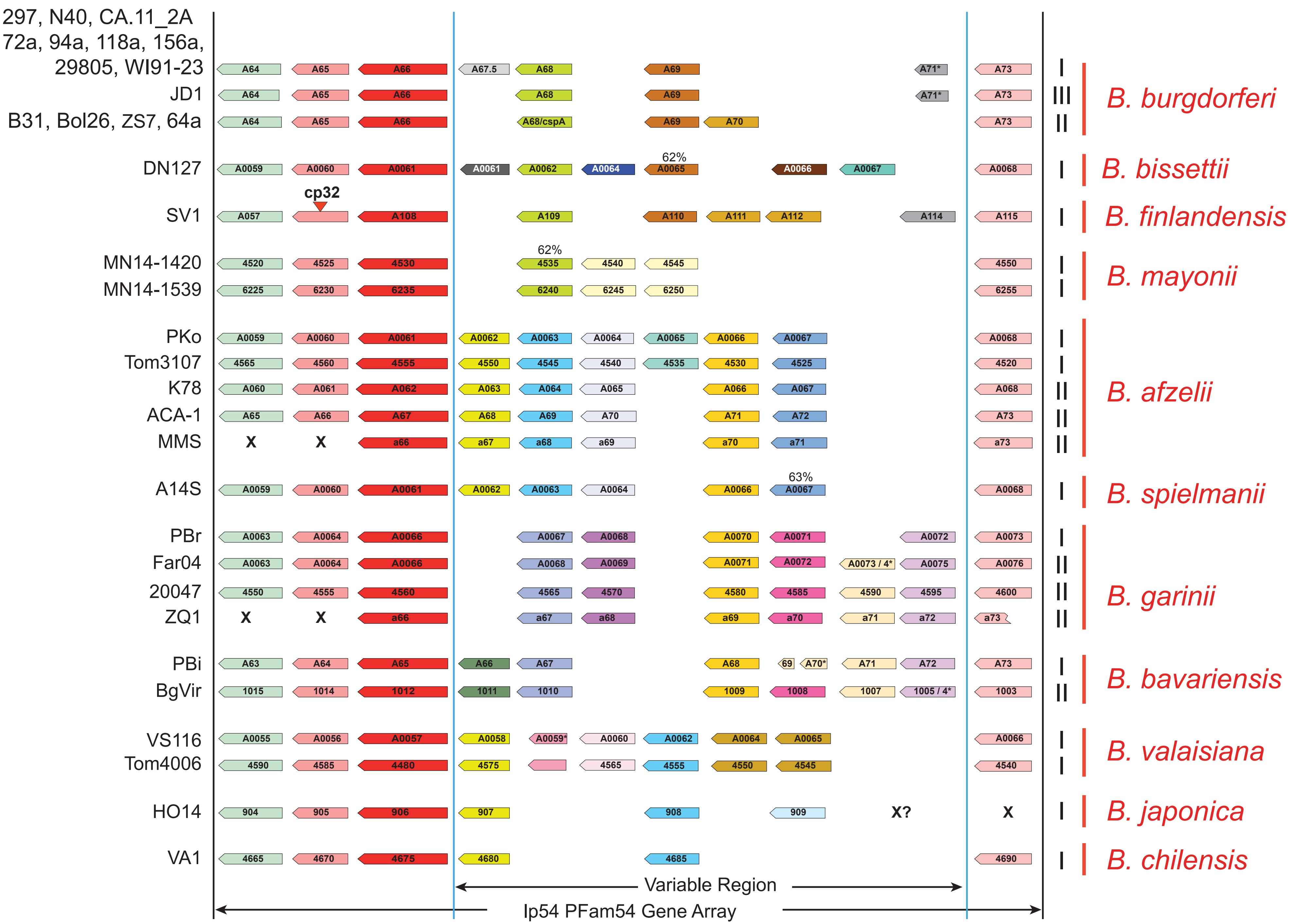

297, N40, CA.11_2A 72a, 94a, 118a, 156a, 29805, WI91-23
A64 A65 A66 A67.5 A68 A69 A71* A73
JD1
A64 A65 A66 A68 A69 A71* A73
B31, Bol26, ZS7, 64a
A64 A65 A66 A68/cspA A69 A70 A73
B. burgdorferi
DN127
A0059 A0060 A0061 A0061 A0062 A0064 62% A0065 A0066 A0067 A0068
B. bissettii
cp32
SV1
A057 A108 A109 A110 A111 A112 A114 A115
B. finlandensis
MN14-1420
4520 4525 4530 62% 4535 4540 4545 4550
MN14-1539
6225 6230 6235 6240 6245 6250 6255
B. mayonii
PKo
A0059 A0060 A0061 A0062 A0063 A0064 A0065 A0066 A0067 A0068
Tom3107
4565 4560 4555 4550 4545 4540 4535 4530 4525 4520
K78
A060 A061 A062 A063 A064 A065 A066 A067 A068
ACA-1
A65 A66 A67 A68 A69 A70 A71 A72 A73
MMS
X X a66 a67 a68 a69 a70 a71 a73
B. afzelii
A14S
A0059 A0060 A0061 A0062 A0063 A0064 A0066 63% A0067 A0068
B. spielmanii
PBr
A0063 A0064 A0066 A0067 A0068 A0070 A0071 A0072 A0073
Far04
A0063 A0064 A0066 A0068 A0069 A0071 A0072 A0073 / 4* A0075 A0076
20047
4550 4555 4560 4565 4570 4580 4585 4590 4595 4600
ZQ1
X X a66 a67 a68 a69 a70 a71 a72 a73
B. garinii
PBi
A63 A64 A65 A66 A67 A68 69 A70* A71 A72 A73
BgVir
1015 1014 1012 1011 1010 1009 1008 1007 1005 / 4* 1003
B. bavariensis
VS116
A0055 A0056 A0057 A0058 A0059* A0060 A0062 A0064 A0065 A0066
Tom4006
4590 4585 4480 4575 4565 4555 4550 4545 4540
B. valaisiana
HO14
904 905 906 907 908 909 X? X
B. japonica
VA1
4665 4670 4675 4680 4685 4690
B. chilensis
I
III
II
Variable Region
lp54 PFam54 Gene Array

Lyme Agent *Borrelia* Chromosome Sequence Left Ends

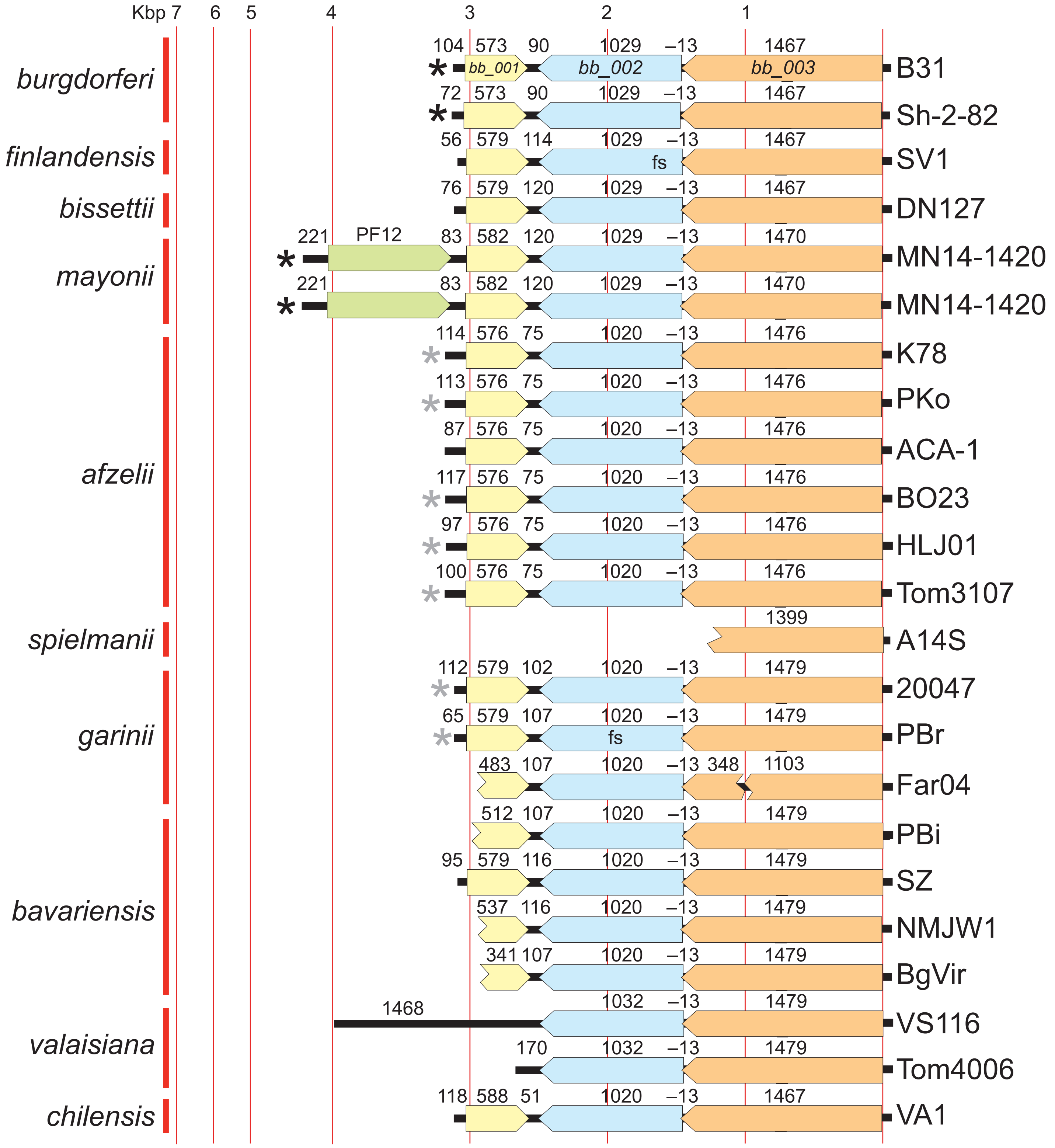

Figure S5B

Lyme Agent *Borrelia* Chromosome Sequence Right Ends

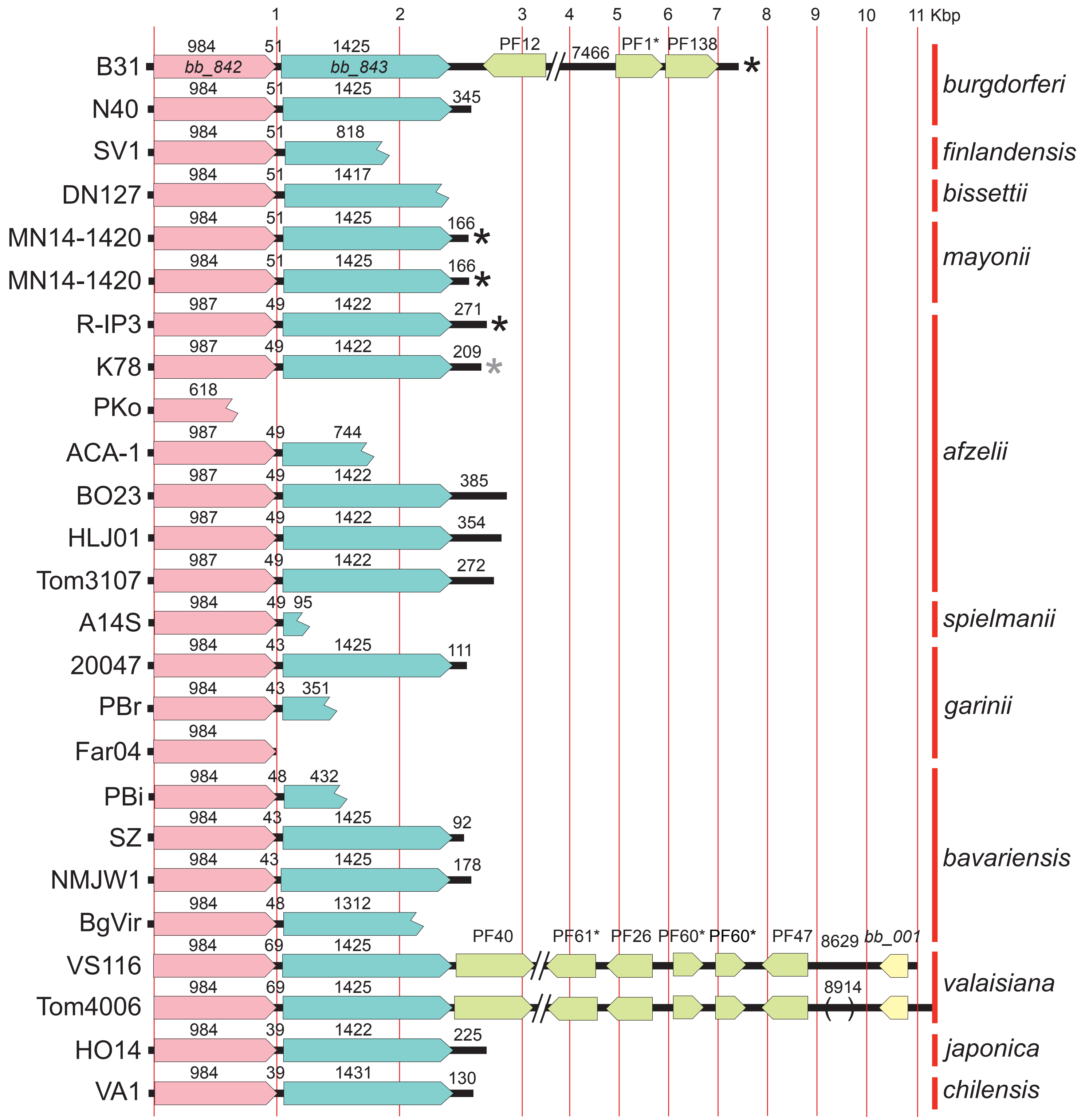

cp9

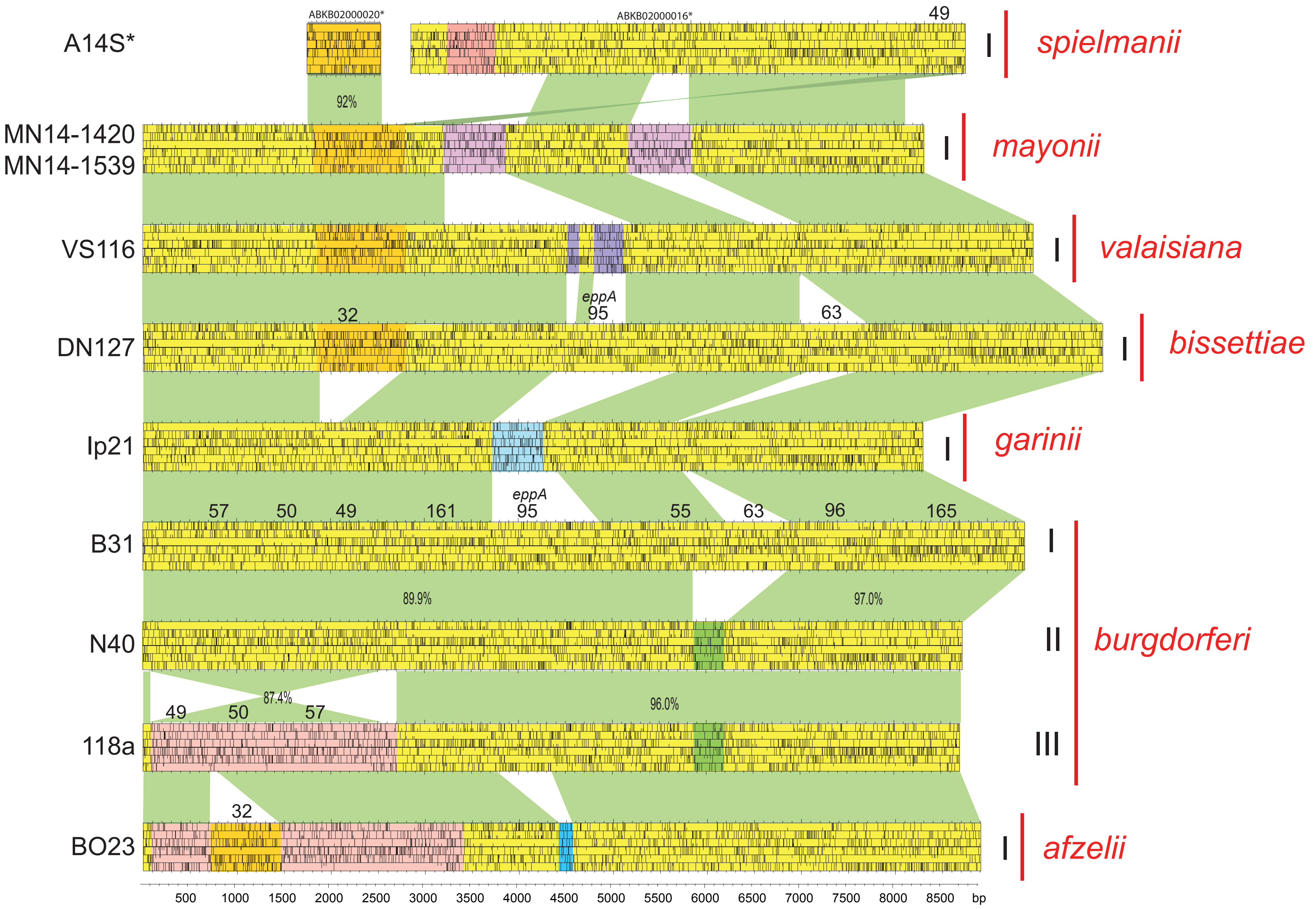

* A14S cp9 sequence not "closed"

Figure S7

cp32-like contigs in the *B. spielmanii* A14S genome

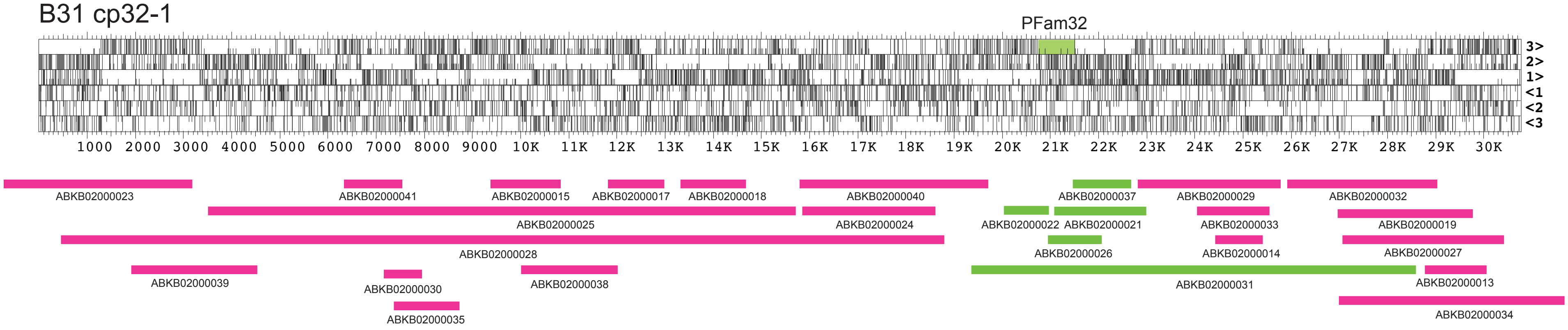

14S contigs that encode PFam32 proteins (green horizontal lines)

ABKB02000022 N-term 186 AA of cp32-12 type PFam32 protein
ABKB02000021 C-term 128 C-term cp32-12 type PFam32 protein
ABKB02000037 C-term 37 AA of probable cp32-10 PFam32 type protein (best match to lp32-10)
ABKB02000031 whole cp32-5 type PFam32 protein
ABKB02000026 whole cp32-3 type PFam32 protein

Figure S8

Rearrangements in cp32-like plasmids in NBu-BbsI genomes

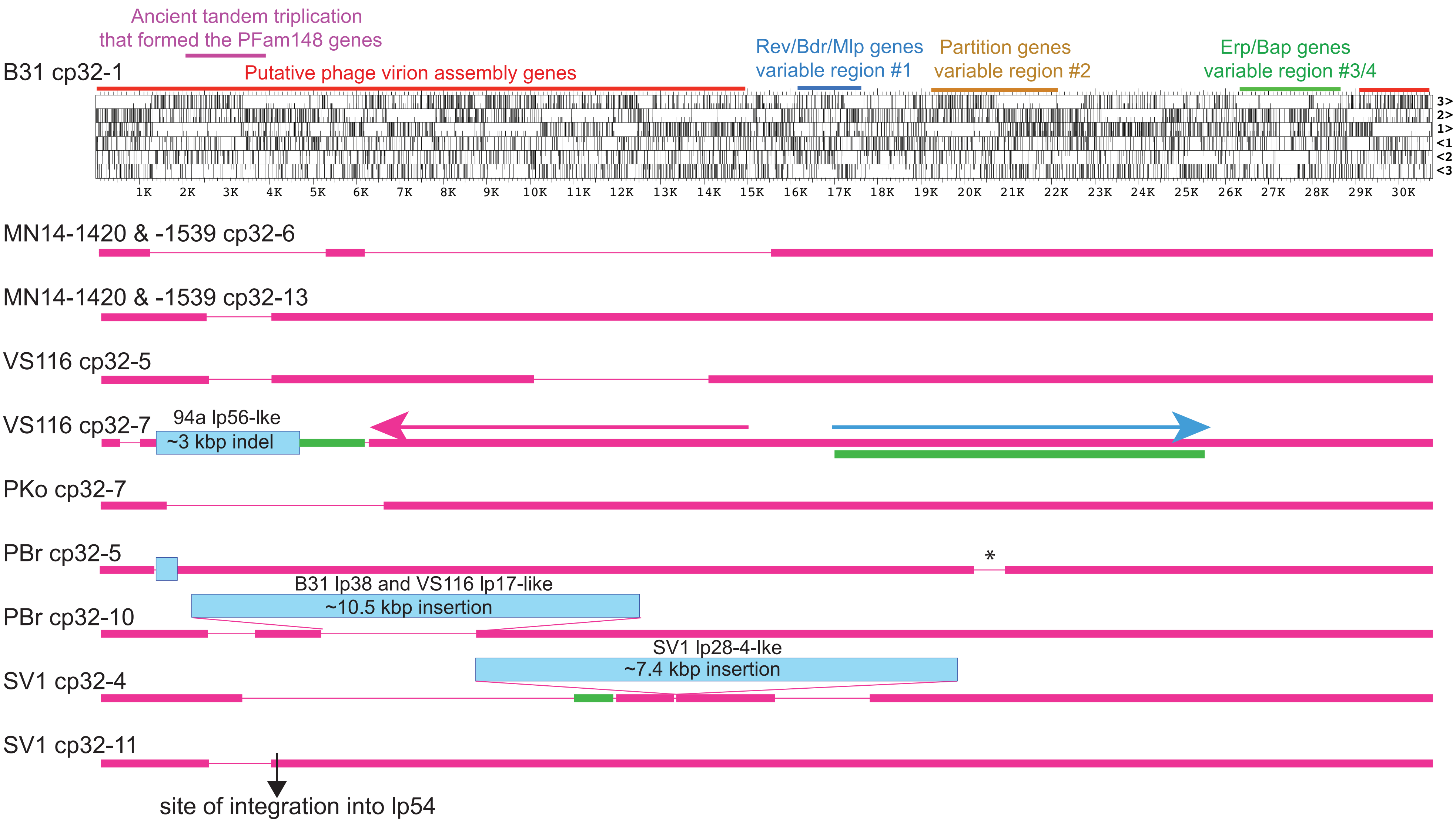

* short deletion truncates BGAPBR_V0033, the PFam49 gene in the partition gene cluster

Figure S9

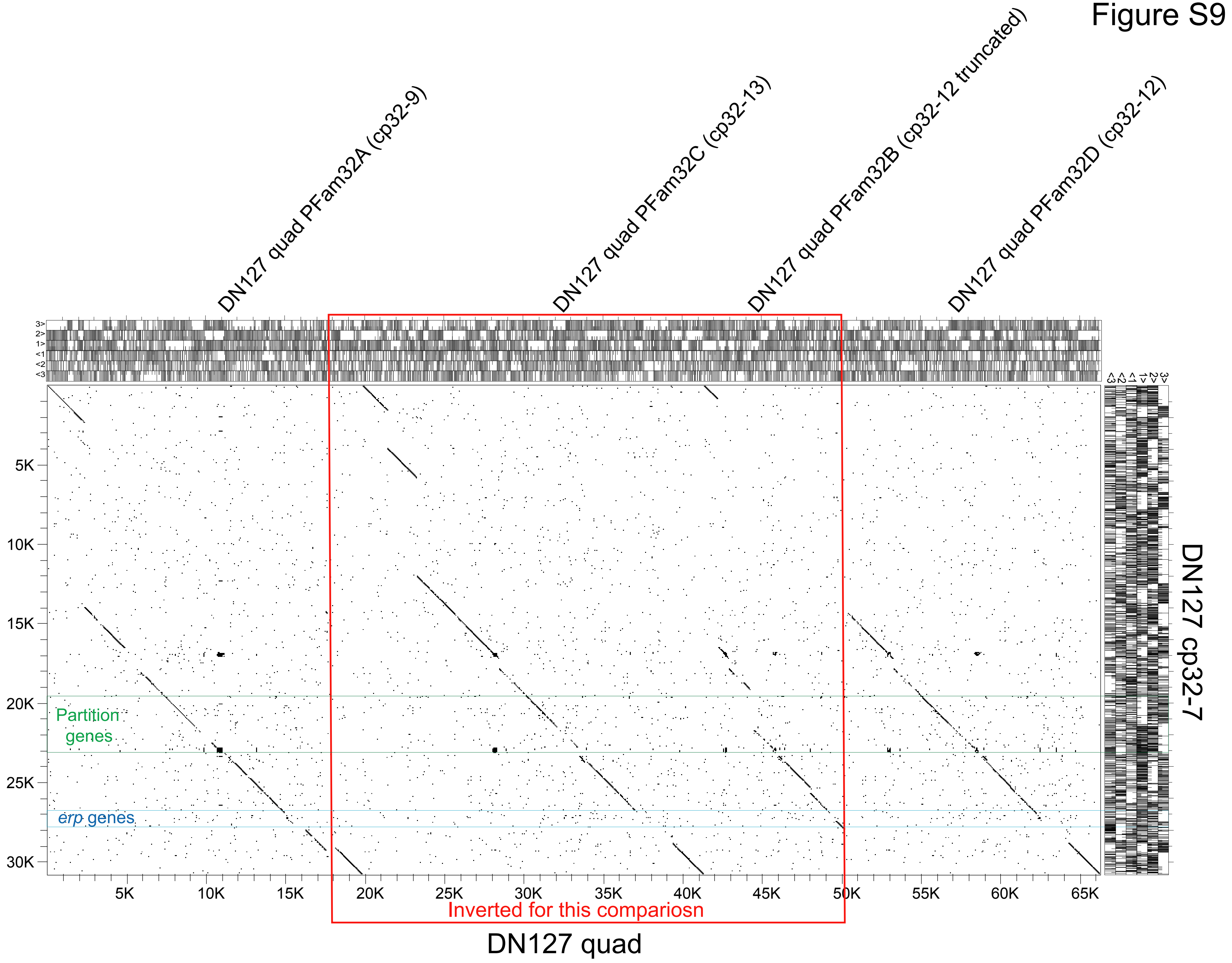
DN127 quad PFam32A (cp32-9)
DN127 quad PFam32C (cp32-13)
DN127 quad PFam32B (cp32-12 truncated)
DN127 quad PFam32D (cp32-12)
3>
2>
1>
<1
<2
<3
5K
10K
15K
20K
25K
30K
Partition genes
erp genes
5K
10K
15K
20K
25K
30K
35K
40K
45K
50K
55K
60K
65K
Inverted for this compariosn
DN127 quad
DN127 cp32-7

Figure S10A

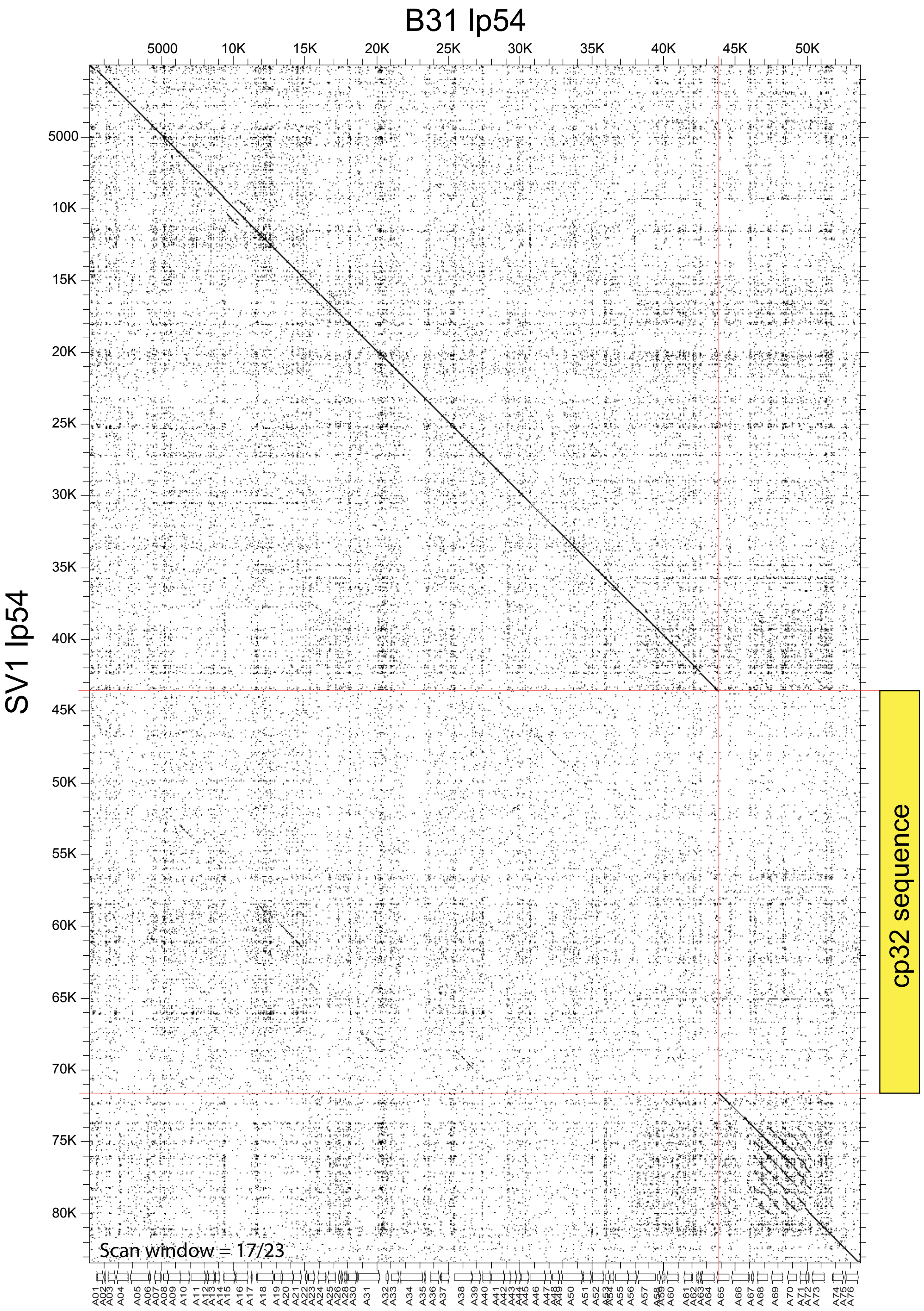

Figure S10B

Putative crossover lp54-cp32 point (^) in *B. finlandensis* SV1 fusion plasmid

```
GGTCGTTTAGCTTTTCTG^TATTGTATTGTAGCT lp54
-------|-----|----  |--|-----------
CAAAAGCTGTGGCTAATA^TTATAGGAGAAGTTA cp32
```

Figure S11

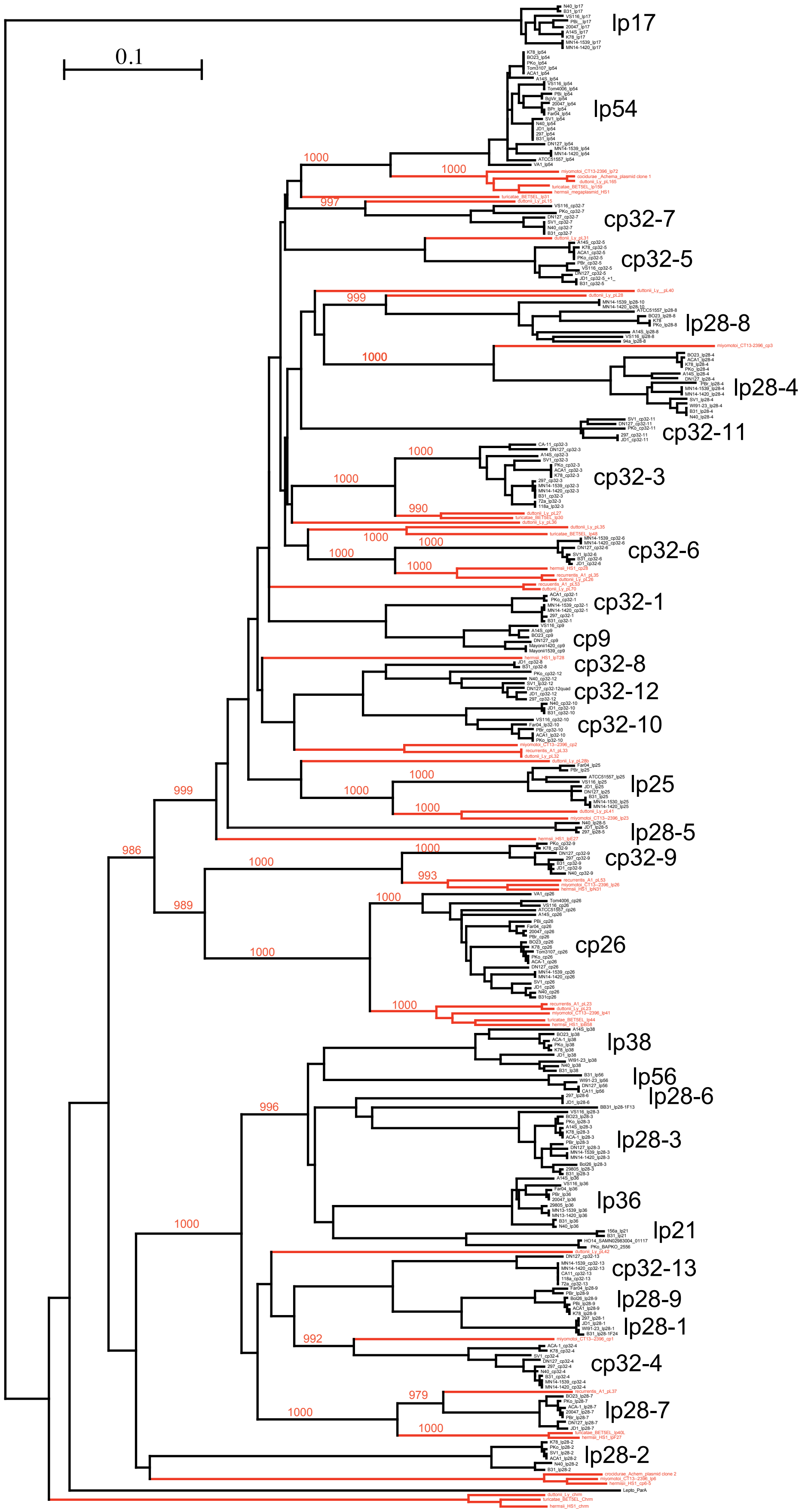

0.1
lp17
lp54
1000
1000
997
cp32-7
cp32-5
999
lp28-8
1000
lp28-4
cp32-11
1000
1000
cp32-3
990
1000
1000
1000
cp32-6
1000
cp32-1
cp9
cp32-8
cp32-12
cp32-10
999
1000
1000
lp25
1000
lp28-5
986
1000
1000
cp32-9
993
989
1000
1000
cp26
1000
lp38
lp56
lp28-6
996
lp28-3
lp36
1000
lp21
cp32-13
lp28-9
lp28-1
992
cp32-4
979
1000
lp28-7
1000
lp28-2
